# Uncovering population structure in the Humboldt penguin (*Spheniscus humboldti*) along the Pacific coast at South America

**DOI:** 10.1371/journal.pone.0215293

**Published:** 2019-05-10

**Authors:** Gisele P. M. Dantas, Larissa R. Oliveira, Amanda M. Santos, Mariana D. Flores, Daniella R. de Melo, Alejandro Simeone, Daniel González-Acuña, Guillermo Luna-Jorquera, Céline Le Bohec, Armando Valdés-Velásquez, Marco Cardeña, João S. Morgante, Juliana A. Vianna

**Affiliations:** 1 PPG Biologia de Vertebrados, Pontifícia Universidade Católica de Minas Gerais, Belo Horizonte, Brazil; 2 Instituto de Biologia, Universidade de São Paulo (IB-USP), São Paulo, Brazil; 3 Universidade do Vale do Rio dos Sinos (UNISINOS), São Leopoldo, Rio Grande do Sul, Brazil; 4 Universidade Federal de Minas Gerais (UFMG), Belo Horizonte, Brazil; 5 Universidad Andrés Bello, Facultad de Ecología y Recursos Naturales, Santiago, Chile; 6 Universidad de Concepción, Facultad de Ciencias Veterinarias, Chillán, Chile; 7 Departamento de Biología Marina, Facultad de Ciencias del Mar, Universidad Católica del Norte, Coquimbo, Chile; 8 Université de Strasbourg, Centre National de la Recherche Scientifique (CNRS); Institut Pluridisciplinaire Hubert Curien (IPHC), Strasbourg, France; 9 Département de Biologie PolaireCentre Scientifique de Monaco (CSM), Principality of Monaco, Monaco; 10 Centro de Investigación para el Desarrollo Integral y Sostenible (CIDIS) and Facultad de Ciencias y Filosofía, Universidad Cayetano Heredia, Lima, Perú; 11 Programa Punta San Juan (CSA-UPCH), Universidad Peruana Cayetano Heredia, Lima, Perú; 12 Departamento de Ecosistemas y Medio Ambiente, Facultad de Agronomía e Ingeniería Forestal, Pontificia Universidad Católica de Chile, Santiago, Chile; Charles Darwin University, AUSTRALIA

## Abstract

The upwelling hypothesis has been proposed to explain reduced or lack of population structure in seabird species specialized in food resources available at cold-water upwellings. However, population genetic structure may be challenging to detect in species with large population sizes, since variation in allele frequencies are more robust under genetic drift. High gene flow among populations, that can be constant or pulses of migration in a short period, may also decrease power of algorithms to detect genetic structure. Penguin species usually have large population sizes, high migratory ability but philopatric behavior, and recent investigations debate the existence of subtle population structure for some species not detected before. Previous study on Humboldt penguins found lack of population genetic structure for colonies of Punta San Juan and from South Chile. Here, we used mtDNA and nuclear markers (10 microsatellites and RAG1 intron) to evaluate population structure for 11 main breeding colonies of Humboldt penguins, covering the whole spatial distribution of this species. Although mtDNA failed to detect population structure, microsatellite loci and nuclear intron detected population structure along its latitudinal distribution. Microsatellite showed significant *R*_*st*_ values between most of pairwise locations (44 of 56 locations, *R*_*st*_ = 0.003 to 0.081) and 86% of individuals were assigned to their sampled colony, suggesting philopatry. STRUCTURE detected three main genetic clusters according to geographical locations: i) Peru; ii) North of Chile; and iii) Central-South of Chile. The Humboldt penguin shows signal population expansion after the Last Glacial Maximum (LGM), suggesting that the genetic structure of the species is a result of population dynamics and foraging colder water upwelling that favor gene flow and phylopatric rate. Our findings thus highlight that variable markers and wide sampling along the species distribution are crucial to better understand genetic population structure in animals with high dispersal ability.

## Introduction

In species with high dispersal ability and no geographical barriers in their distribution, it is expected found low genetic population structure. For instance, weak or no population genetic structure has been frequently recorded for seabird species along the Atlantic coast of South America (e.g. Kelp gull, *Larus dominicanus* [1,2]; Magellanic penguin, *Spheniscus magellanicus* [3]; South-American tern, *Sterna hirundinacea* [4]), along the Pacific coast of South America (e.g. Peruvian pelican, *Pelecanus thagus* [5]), and around Antarctica (Emperor penguin, *Aptenodytes forsteri*, [6, 7]; Adélie penguin, *Pygoscelis adelia* [8]; *P*. *antarticus* Chinstrap penguin [9]). Therefore, relative importance of factors that influence the population structure of seabirds have been under debate, such as the presence of physical or non-physical barriers [10,11], the foraging ecology of the species [12], and/or their philopatric behavior [13].

The upwelling hypothesis has been proposed to explain reduced or lack of population genetic structure in seabird species specialized in food resources available at cold-water upwellings, which are regularly influenced by largescale climatic events [12]. For instance, the Southeast Pacific coast is characterized by the Humboldt Current System (HCS) of cool sea surface temperature and high biological productivity. In the HCS, the coastal upwellings provide more than 10% of global fish catch [14], however, these areas are not temporally continuous or spatially uniform: indeed, El Niño Southern Oscillation (ENSO) reduces the upwellings’ intensity leading to the warming of surface waters, reducing productivity [15, 16, 17, 18], and affecting the survival and dispersal of fishes, seabirds and marine mammals [19, 20, 21]. Thus, during El Niño events, adult seabirds disperse long distances to find new productive upwelling areas to forage and colonize new area, according to the availability of breeding grounds, increasing gene flow and consequently reducing population genetic structure. The weak population genetic structure and high genetic diversity of Humboldt Current endemic seabirds can be explained by the upwelling hypothesis [5], such as described for Peruvian pelicans, Peruvian boobies [12], and also for Humboldt penguins [22].

Another hypothesis to explain reduced population genetic structure is the effect of Pleistocene glaciations, which is frequently proposed for seabirds from South America [23] and from the North Hemisphere [24, 25]. During the Last Glacial Maximum (LGM), the southern portion of the Pacific coast was covered by an ice sheet [26, 27]. However, in Chile, the region between 33°S and 46°S was considered to have been climatically stable [28]. Thus, distinct climate conditions throughout the Pacific coast could play an important role in defining the demographic history of populations, affecting species distribution, and leaving a genetic signature on populations. Therefore, low genetic structure could reflect the signature of population expansion after the LGM associated with high population size (Ne), thus recent gene flow would not be easily detected.

Gene flow among populations derives from contemporary and historical factors. However, detecting population genetic structure in species with large effective population size is a challenge, since variation in allele frequencies is masked by genetic drift that is inversely proportional to population size [29]. There is also debate on the power of algorithms of clustering to detect genetic structure in species with large population [6, 30, 31, 32]. For instance, Chinstrap penguins showed weak genetic population structure and a pattern of isolation by distance (IBD) when evaluating four breeding colonies [33] at southernmost Western Antarctic Peninsula (WAP), but no differentiation from the distant Bouvet Island and 11 WAP locations [9]. The number of molecular markers, loci, sample size, and the number of locations across the species distribution might be important to fully understand these patterns. Therefore, the detection of population genetic structure for seabirds from HCS may not only reject the upwelling hypothesis, but may propose new hypotheses to explain the new patterns of species across the region.

Penguins are monogamous seabirds, with intense biparental care, philopatric behavior, high capacity of dispersion, and specialist diet [34]. The Humboldt penguin (*Spheniscus humboldti*) is an HCS specialist, widely distributed along the Pacific coast of South America, from La Foca Island (05°12’S; 81°12’W) in Peru to Metalqui Island (42°12’S; 74°09’W) in Chile [35]. There are records that Humboldt penguins have been seen at Guafo Island (43°36’S) in a mixed-species colony, however there is no report of breeding activity [36]. On the southern limit of its distribution, there is information about hybridization between Humboldt and Magellanic penguins in mixed-species colonies [37]. The current global population size estimation is *ca*. 32,000 to 36,982 breeding adults, and the Humboldt Penguin is listed as Vulnerable by International Union for Conservation of Nature (IUCN) due to population size reductions attributed to exploitation or habitat alteration, as well as the effect of ENSO events [38, 39].

Migration rates among colonies are not well known, however there is evidence of individuals from Pan de Azucar colony migrating over 600 km, and birds from Puñihuil were found over 1,000 km northwards from their original colonies [40, 41]. In addition, it was proposed that during ENSO events, Humboldt penguin abundance and distribution might have been shifted southward, causing the reduction of populations in the North (e.g. Punta San Juan, Peru) and an increase in some colonies in Chile, such as Chañaral Island [39]. Intense migration rate has been corroborated by low genetic structure estimated among some colonies of Humboldt penguins [22].

The present study aims to evaluate population genetic structure of the Humboldt penguin, testing upwelling and glaciation hypotheses to explain lack or reduced population structure as previously proposed for this species, and to investigate the potential contribution of choice of molecular markers for population genetic structure of the species along the HCS. To achieve these goals, we (1) characterize the genetic diversity and geographical structure across the entire species range, (2) determined the effects of historical climate changes over the species demography, and (3) evaluated if there is sex-biased philopatry and dispersal, bringing light to the questions about low structure recorded on several seabird´ species.

## Material and methods

### Ethics statement

This study was carried out in strict accordance with the recommendations in the Guide for the Care and Use of Animals at research of the National Council of Animal Experiments from Brazil and Bioethics Guideline from CONICYT (Comisíon Nacional de Investigacion Cientifica y Tecnologica) del Chile. The protocol was approved by the Committee on the Ethics of Animal Experiments of the Pontificia Universidade Catolica from Minas Gerais (Protocol Number: 27–2016) and by the Committee on the Ethics of Animal Experiments of the Pontificia Universidad Catolica del Chile. Samples were obtained under Subpesca—Corporación Nacional Forestal del Chile (CONAF), Direccíon General Florestal y de Fauna Silvestre (DGFFS) of the Peruvian Nation Protected Areas Agency (SERNANP 001088) and (Instituto Brasileiro do Meio Ambiente e Recursos Naturais (CITES IBAMA 10BR005149DF; 10BR005120).

### Sampling

**Fig 1:**
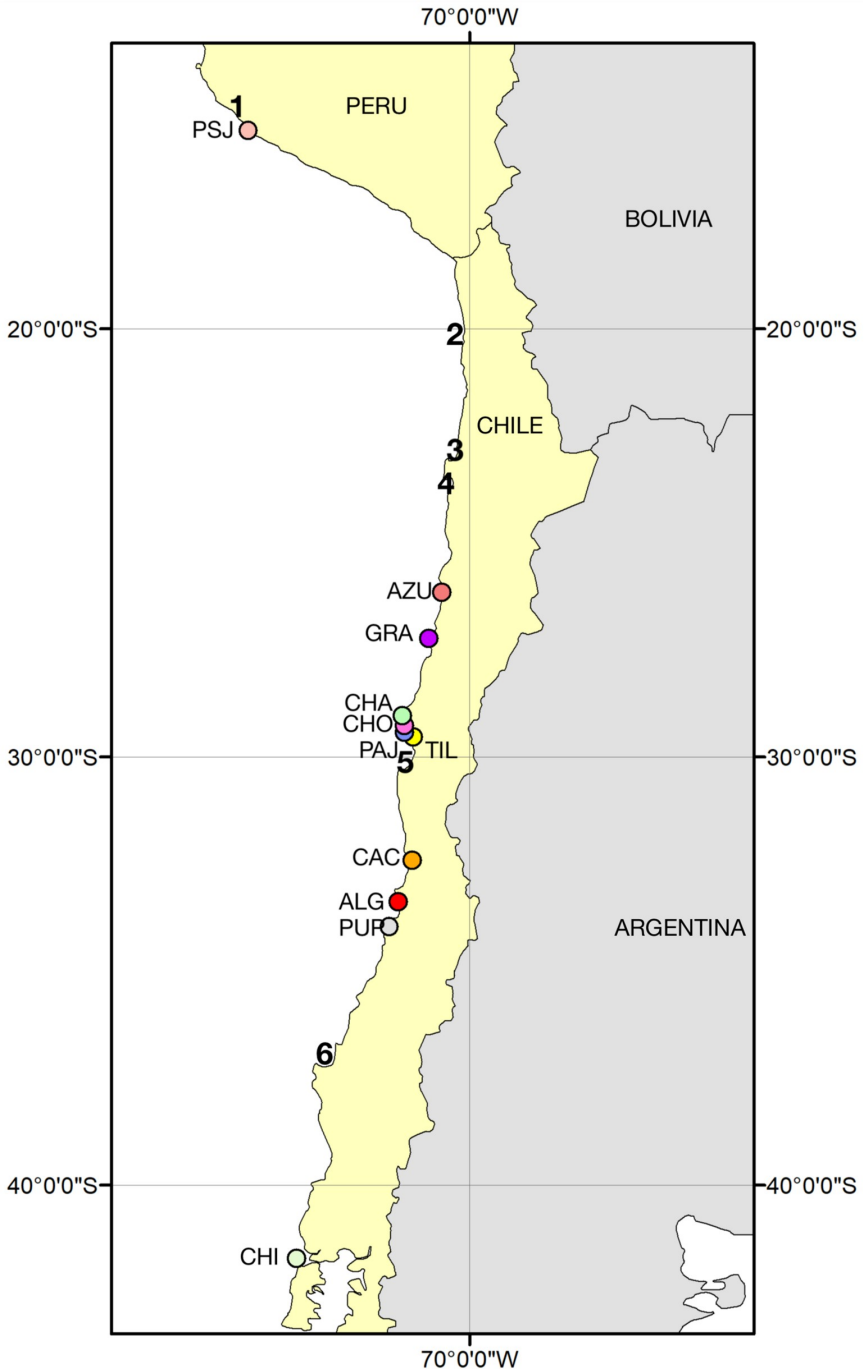
Map of South America showing sampling locations of the Humboldt penguin: CHI (Chiloé), PUP (Pupuya), ALG (Algarrobo), CAC (Cachagua), TIL (Tilgo), PAJ (Pajaros), CHO (Choros), CHA (Chañaral), GRA (Isla Grande), AZU (Pan de Azucar), and PSJ (Punta San Juan).

Blood samples for genetic analysis were collected during penguin breeding seasons between 2005 and 2013. We collected a total of 487 samples from adult Humboldt penguins from 11 breeding colonies distributed along its entire distribution range in Peru and Chile (Fig 1). Penguins were captured quietly with a noose pole 1.5 m in length used to lead the penguins out of their nests, and they were hold manually. The heads of captured penguins were covered by one mask to reduce the visual stress. Approximately 500 μl (microliters) of blood samples were obtained from the internal metatarsal vein or the brachial vein, using a 23G needle and 3 mL syringe and stored in 96% ethanol P.A. for genetic analysis. To avoid re-sampling, penguins were marked temporarily with water-resistant color markers, with exception in Punta San Juan colony where penguins already had flipper bands. DNA was isolated for genetic analysis using standard phenol-chloroform extraction protocols followed by ethanol precipitation and DNA resuspension in sterile water [42].

### Molecular sexing

Sex was determined by Polymerase chain reaction (PCR), using primer pairs P_2_ and P_8_ [43], prepared to 10 μL volume containing 50 ng of total DNA, 1X of *Taq* buffer, 200 μM of each dNTP, 3.5 mM MgCl_2_, 0.5 μM of each primer, and 0.5 U of *Taq DNA* polymerase. Amplifications were performed with an initial step at 94°C for 4 min, 53–55°C for 30 s, and 1 min at 72°C, followed by 40 cycles of 30 s at 92°C, 50°C for 30 s, and 45 s at 72°C, followed by a final extension of 7 min at 72°C. The PCR amplifies regions of the CHD1 gene found on the sex chromosomes [43]. Gender identification was based on the number of bands for a given sample visualized on 3% agarose gel. Males have a single band that corresponds to the intron on Z chromosomes, whereas females have two bands, corresponding to introns on the ZW chromosomes that showed distinct size.

### Microsatellite genotyping

We genotyped 13 microsatellite loci developed for *Spheniscus* [22, 44, 45]. PCR amplification and genotyping procedures were performed using fluorescently labeled primers (Thermofisher, São Paulo, Brazil). PCR were conducted separately for each locus in a 10 μL volume containing 20–40 ng of total DNA, 1X of *Taq* buffer, 200 μM of each dNTP, 0.5 μM of each primer, and 0.5 U of *Taq DNA* polymerase. Amplifications were performed with an initial step at 95°C for 4 min and 37 cycles of 30 s at 94°C, 30 s at annealing temperature according to each locus (S1 Table), and 1 min at 72°C, followed by a final extension of 10 min at 72°C. PCR products were diluted 5X (1:5). A volume of 1.5 μL diluted PCR from FAM labeled and 3.0 μL from HEX labeled was then suspended in 7.75 μL HiDi Formamide (Applied Biosystems, Foster City, CA) with 0.25 of GeneScan 500 ROX size standard, and analyzed on ABI 3500 (Applied Biosystems, Foster City, CA). The fragments were genotyped using GeneMapper v.4 software (Applied Biosystems, Foster City, CA).

Genotyping error or null alleles were tested for each colony using the program MicroChecker [46]. Hardy-Weinberg and linkage equilibrium for each locus within each colony, as well as all colonies and all loci together were evaluated in GenAlEx [47]. Allele frequencies, allelic richness, observed and expected heterozygosity were estimated for each colony using GenAlEx. Differences in the distribution of genetic variation among colonies were analyzed by R_ST_ between colonies using ARLEQUIN v.3.1 [48].

Possible correlations between the indices of genetic differentiation and geographic distances between all pairs of colonies were tested using the Mantel test, which was performed with Mantel Non-parametric Test Calculator v.2.0 [49] using 10,000 randomizations. The distances between the pairwise colonies (islands) were calculated from geographic coordinates by the program GPS coordinate (https://gps-coordinates.org/distance-between-coordinates.php) considering a Euclidean distance.

Bayesian clustering analyses was performed to estimate the optimal number of genetic clusters (K) using STRUCTURE v2.3.3 [50]. To test for population genetic structure without prior knowledge of sampling locations, we estimated the posterior probability of the data fitting the hypothesis of K clusters [Pr(X|K)], where K is the number of putative populations. STRUCTURE was run using the admixture and noadmixture model, without localities as priors and assuming uncorrelated allelic frequencies. Preliminary runs, testing from K = 1 to K = 10, were repeated 10 times, each run had 100,000 cycles of burn-in and 10,000 cycles of MCMC. STRUCTURE HARVESTER was used to infer the simplest model to genetic population structure performing Evanno’s method (ΔK) [51]. Also, a Discriminant Analysis of Principal Components (DAPC) was carried out to determine the number of cluster of genetically related individuals, with a non-Bayesian approach. DAPC uses sequential *K*-means and model selection to identify genetic cluster [52]. For this, adegenet package in R [53] was used, retaining all principal components.

Assignment of each individual to their reference putative population was evaluated for microsatellite data using GeneClass [54], employing the likelihood method based on allele frequencies [55], as well as a Bayesian approach [56]. The probability that each individual was assigned to a candidate population was estimated using a Monte Carlo resampling method [57] (number of simulated individuals = 10,000; type I error = 0.01, applying rejection threshold of 0.05). The same program was used to detect first generation migrants employing the Bayesian criterion [56] applying the Monte Carlo resampling method [57] with 10.000 simulated individuals and an alpha of 0.01. We used the highest likelihood value among all available populations (L = L_home/L_max). Philopatric rate and migrate rate difference between sexes were tested by Mann-Whitney non-parametric test using Bioestat Software [58]. Gene flow between population also were estimated through coalescence-based maximum likelihood (LMAX) method implemented in MIGRATE-n 3.2.6 [59], considering geographical distance. MIGRATE assumes an n-island model at mutation migration-drift equilibrium with values of M and θ constant over time. The Brownian motion model was used as an approximation of the stepwise mutation model, and 10 following initial trials, search criteria for the MCMC sampler were set to 20 short chains of 20 000 steps and 3 long chains of 200 000 steps.

Deviations from mutation/drift equilibrium were tested with the program BOTTLENECK [60, 61]. Three models of microsatellite evolution were tested: infinite allele model (IAM), two-phase model (TPM), and the stepwise mutation model (SMM). The TPM is the most realistic model for microsatellite mutation because it assumes mainly stepwise mutations with some multi-step mutations [62]. Parameters for the TPM were set at 90% single step mutations, as suggested for microsatellite data [60, 61].

### Mitochondrial DNA and nuclear DNA sequences

The mtDNA Control region was amplified with primers D-loop C and D [63]. The PCR reaction (10 μL) contained 20 ng of DNA, 1x of *Taq* buffer, 200 μM of each dNTP, 1.0 μM of each primer, and 0.5 U of *Taq* polymerase. Amplifications were performed with an initial step at 94°C for 2 min and 35 cycles of 30 s at 94°C, 40 s at 62°C and 90 s at 72°C, followed by final extension of 10 min at 72°C. PCR products were cleaned by precipitation using 20% Polyethylene Glycol with 2.5M NaCl. Sequences were obtained on an ABI 3500.

RAG-1 (Recombination activating gene 1) was amplified with primers RAG17 and RAG22 [64]. The PCR reaction as prepared for 10 μL as before (D-loop) Amplifications were performed with an initial step at 94°C for 2 min and 35 cycles of 30 s at 94°C, 40 s at 59°C and 90 s at 72°C, followed by final extension of 10 min at 72°C. PCR products were cleaned and sequenced as before.

Sequences were visualized using ChromasLite 2.1 (www.technelysium.com.au). The alignments were adjusted by manually in Bioedit v.5.06 [65]. A Bayesian approach run with the program PHASE [66] was used to identify haplotypes of heterozygotes in the nuclear intron: this program reconstructs the haplotype as implemented in DNAsp v.5.10.01 software [67].

Descriptive analyses, including haplotype diversity (h), nucleotide diversity (π), and theta (θ), were done in DNAsp v.5.10.01 [67]. We used the Network software version 4.6 (www.fluxus-technology.com) with median joining method [68] to draw relationships among haplotypes. Additionally, we calculated Tajima’s [69] and Fu’s [70] statistics to test bias from neutrality in DNAsp v.5.10.01 [67]. We selected these statistical tests due to their power to detect population expansion scenarios in specific sampling conditions and with specified population expansion rate, time since the expansion, sample size and number of segregating sites [71].

We used AIC as implemented in the software JModeltest [72], to select the best-fit evolutionary model. The evolutionary model selected for control region was T92+G with a discrete gamma distribution (α = 0.06), and JC for RAG1 region. These evolutionary models were used in Bayesian Skyline Plots to analyze population size dynamics through time [73], implemented in BEAST 1.6.1 [74]. We performed runs of 200,000,000 steps, logged every 5,000 steps, and burn-in of 20,000,000. For BEAST analysis, we considered the mutation rate of 0.86 substitution/site/myrs to D-loop (HVRI) described for the Adélie penguin [75] and 1.9 X 10^−3^ substitution/site/myrs to RAG1 [76]. To evaluate the convergence of parameters between runs and the performance of analysis (ESS values > 200), we used TRACER 1.7.5 (http://beast.bio.ed.ac.uk/Tracer) [74]. To check the level of population genetic structure among localities, we performed an analysis of molecular variance (AMOVA) with two hierarchical levels using ARLEQUIN 3.5 [46].

## Results

### Genetic diversity

The Humboldt penguin showed high genetic diversity for all markers in all colonies. For microsatellites, the number of alleles found in each locus ranged from eight (locus Sh2Ca58) to 23 (both Sh2Ca40), averaging 15.89 over all loci (S1 Table). Private and rare alleles were found in almost all breeding colonies, except for Pupuya and Isla Grande de Atacama (frequencies of 0.004 to 0.056).

The analyses performed by Microchecker showed evidence for null alleles at locus M1-11 in seven breeding colonies (Algarrobo, Cachagua, Tilgo, Pajaros, Choros, Chañaral and Punta San Juan) and out of Hardy-Weinberg Equilibrium (HWE), thus it was excluded from population analysis. Loci Sh2Ca58, Sh2Ca12 and Sh2Ca9 were also excluded from population analysis due genotype proportions deviating from H-W expectations (S2 Table). Thus, the population analysis was conducted with 10 microsatellite loci. Humboldt penguin breeding colonies through the Pacific coast from South America showed relatively high levels of genetic diversity, with mean heterozygosity 0.72 ± 0.014 (Table 1). The minimum value observed was from Algarrobo Island (H_o_ = 0.66) and the maximum from Tilgo (H_o_ = 0.76).

**Table 1:** Summary statistics of Humboldt penguins based on the 13 microsatellites: Sample size (n), mean number of alleles (N_a_), Shannon Index (I), expected (H_e_) and observed (H_o_) heterozygosity, inbreeding coefficient (*F*_*IS*_), and mitochondrial DNA control region and nuclear RAG1 intron: sample size (n), haplotype diversity (H_d_), nucleotide diversity (π) and Neutrality test of Fu’s *F*_*s*_ (*F*_*s*_), Tajima'*D* (*D*) with respective probability (p). In bold, values that were significant for *Fs* (p < 0.02) and *D* (p < 0.05). Population reference: CHI (Chiloé), PUP (Pupuya), ALG (Algarrobo), CAC (Cachagua), TIL (Tilgo), PAJ (Pajaros), CHO (Choros), CHA (Chañaral), GRA (Isla Grande), AZU (Pan de Azucar), PSJ (Punta San Juan).

*Location*	*Code*	*Geog*.	*Microsatellite*						*D-loop*					*RAG1*				
		*position*	*N*	*N*_*a*_	*I*	*H*_*o*_	*H*_*e*_	*F*_*IS*_	*N*	*H*_*d*_	*π*	*Fs*	*D*	*N*	*Hd*	*π*	*Fs*	*D*
Chiloé	CHI	41° 92 S 74°03 W	5	5.07	1.43	0.75	0.71	0	2	1.00	0.00	0.69	0.01	2	0.00	0.00	0.00	0.00
Pupuya	PUP	33°58S 71°53'W	6	4.23	1.25	0.71	0.76	0	2	1.00	0.01	0.69	0.01		-	-	-	-
Algarrobo	ALG	33°23 S 71°41'W	9	6.53	1.61	0.66	0.76	0.127	5	1.00	0.00	0.01	0.01	2	0.00	0.00	0.00	0.00
Cachagua Island	CAC	32°35'S 71°27'W	15	8.23	1.73	0.72	0.78	0.066		-	-	-	-	4	0.66	0.03	3.15	2.12
Tilgo Island	TIL	29°32'S; 71°20'W	50	10.07	1.82	0.76	0.80	0.177	5	1.00	0.00	0.01	0.01	4	0.00	0.00	1.00	0.00
Pájaros Island	PAJ	29°35'S; 71°32'W	76	10.84	1.82	0.71	0.80	0.146	18	0.86	0.01	-2.54	-0.93	10	0.80	0.02	0.33	0.02
Choros Island	CHO	29°16'S 71°32'W	79	11.53	1.89	0.75	0.82	0.147	15	1.00	0.01	0.13	0.01	8	0.85	0.02	-1.00	0.50
Chañaral Island	CHA	29°02'S 71°35'	55	9.84	1.76	0.75	0.79	0.071	2	-	-	-	-	6	0.80	0.01	-0.08	1.03
Grande Island	GRA	27°14'S 70°58'	13	6.46	1.43	0.69	0.66	0	13	0.93	0.01	-3.14	-1.06		-	-	-	-
Pan de Azúcar	AZU	26°09'S 70°41'W	52	8.46	1.6	0.72	0.72	0.003	36	0.91	0.01	**-9.31**	**-1.63**	10	0.82	0.01	-1.08	-0.32
Punta San Juan	PSJ	15°22’S 75°11'W	112	11.84	1.85	0.7	0.77	0.012	70	0.87	0.01	**-5.87**	-1.34	10	0.91	0.02	-1.86	-0.27
	All		463	8.47	1.66	0.73	0.78	0.05		0.89	0.01	**-17.02**	**-1.63**		0.87	0.02	-18.91	-2.04

Analysis of the 401 bp fragment of the D-loop HVRI mtDNA from 175 individual Humboldt penguins showed a total of 37 haplotypes, high haplotype diversity (H_d_ = 0.906) and high nucleotide diversity (π = 0.008). A RAG1 fragment of 876 bp from 54 individuals showed 23 haplotypes, also high haplotype diversity (H_d_ = 0.876) and nucleotide diversity (π = 0.002). These patterns of high genetic diversity were also observed in all colonies analyzed (Table 1).

### Population genetics structure

Genetic variability using AMOVA was found mainly within populations rather than among populations. For microsatellite loci, 96.89% of genetic variability was detected within populations and only 3.11% among populations (p < 0.001), compared to 98.36% within and only 1.64% among populations for mtDNA (p = 0.10), and 78.51% within and 21.49% among populations for RAG1 (p < 0.001).

**Fig 2:**
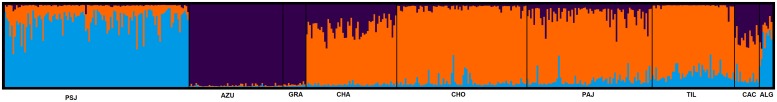
Bayesian STRUCTURE of the Humboldt penguin, delta K = 3, using admixture model. 1- Punta San Juan; 2- Isla Pan de Azucar; 3- Isla Grande de Atacama; 4- Chañaral; 5- Choros; 6- Pájaros; 7- Tilgo; 8- Cachagua; 9- Algarrobo; 10- Pupuya; 11- Chiloé.

**Fig 3:**
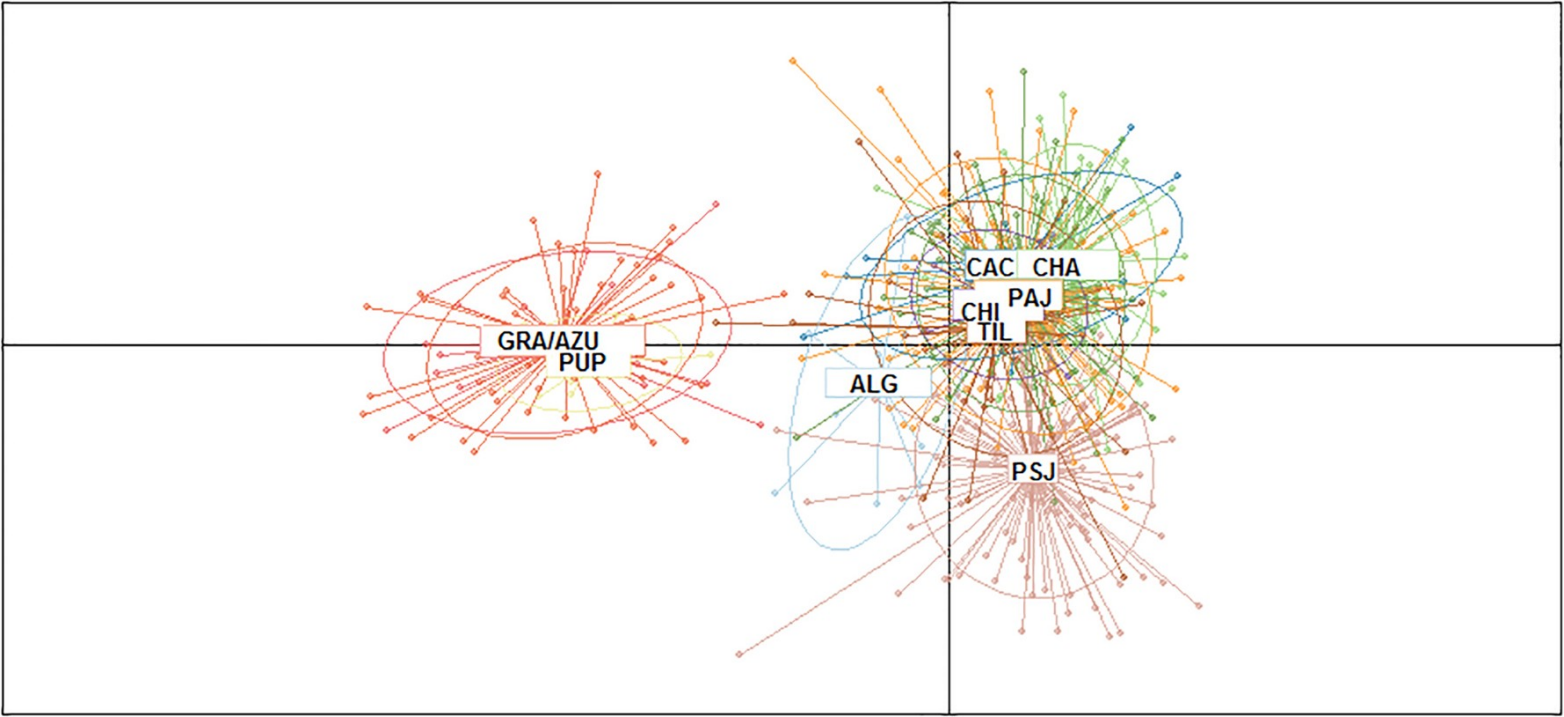
DAPC based on 10 microsatellites of the Humboldt penguin (*Spheniscus humboldti*): CHI (Chiloé), PUP (Pupuya), ALG (Algarrobo), CAC (Cachagua), TIL (Tilgo), PAJ (Pajaros), CHO (Choros), CHA (Chañaral), GRA (Isla Grande), AZU (Pan de Azucar), and PSJ (Punta San Juan).

Bayesian structure analyses of microsatellite data suggested that the existing global population is composed of 3 groups (K = 3) of Humboldt penguins: 1) Punta San Juan, Peru, 2) Pan de Azucar and Isla Grande de Atacama and 3) the remaining locations (Fig 2, S4 Fig and S8 Table). Despite the large geographical distance, Pupuya from the central coast was grouped with the locations from the north (i.e. Pan de Azucar and Isla Grande). However, this is probably due the low sample size from this location. In addition, DAPC estimated the optimal number of cluster to *K* = 2, being one included Pan de Azucar and Isla Grande de Atacama and another included all remaining locations, despite into second group Punta San Juan was slight differentiation (Fig 3). No isolation by distance was identified between all locations, with absence of correlations between geographic and genetic distance (Mantel test; r = 0.04, t = 0.17, p = 0.57).

**Fig 4:**
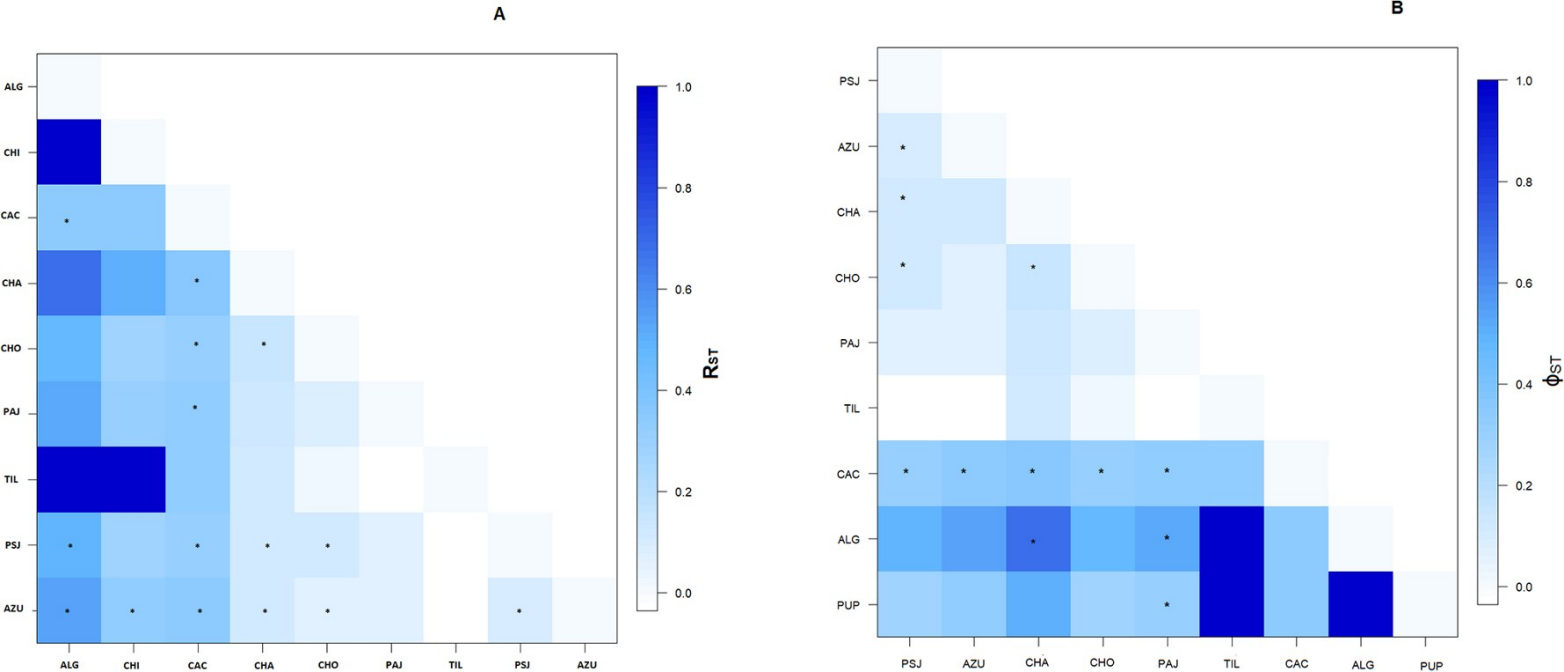
Pairwise R_ST_ based on 10 microsatellites (a), pairwise ϕ_ST_ based on RAG1 (b) of the Humboldt penguin (* p value < 0.05).

Population genetic structure with significant R_ST_ values for microsatellite were observed between the majority of pairwise locations, mainly between the groups detected by STRUCTURE, with significant R_ST_ values ranging between Punta San Juan and the remaining locations of 0.011 to 0.088, or among Pan de Azucar and Isla Grande and remaining locations of 0.001 to 0.158; and within the third group higher values were found between Algarrobo and the remaining locations (0.059 to 0.158), except Pupuya and Chiloé (Fig 4, S3 Table).

RAG1 corroborated with high population structure based on significant ϕ_ST_ for 15 of 36 pairwise comparisons, mainly among Punta San Juan and Pan de Azucar, Chañaral, Choros, Cachagua and Algarrobo; and among Pan de Azucar and Chañaral, Choros, Cachagua and Algarrobo (S4 Table). Cachagua is significantly distinct of almost all colonies, except Tilgo. On the other hand, D-loop region was non-informative, since only one pairwise comparison showed a significant value (S4 Table). In addition, the assignment test indicated that philopatry of the Humboldt penguin is higher than 86% (Table 2).

**Table 2:** Frequency of Humboldt penguin assignment to each population, estimated by maximum likehood based on allele frequencies, where rows represent immigrants and columns represent emigrants. Population reference: CHI (Chiloé), PUP (Pupuya), ALG (Algarrobo), CAC (Cachagua), TIL (Tilgo), PAJ (Pajaros), CHO (Choros), CHA (Chañaral), GRA (Isla Grande), AZU (Pan de Azucar), and PSJ (Punta San Juan).

	ALG	CAC	TIL	PAJ	CHO	CHA	GRA	AZU	PSJ
ALG	1.00	0.00	0.00	0.00	0.00	0.00	0.00	0.00	0.00
CAC	0.07	0.86	0.00	0.00	0.00	0.07	0.00	0.00	0.00
TIL	0.00	0.00	0.90	0.00	0.00	0.04	0.06	0.00	0.00
PAJ	0.00	0.05	0.01	0.89	0.00	0.02	0.03	0.00	0.00
CHO	0.03	0.00	0.01	0.00	0.96	0.00	0.00	0.00	0.00
CHA	0.02	0.00	0.00	0.00	0.00	0.98	0.00	0.00	0.00
GRA	0.00	0.00	0.00	0.00	0.00	0.00	1.00	0.00	0.00
AZU	0.02	0.00	0.00	0.00	0.00	0.00	0.04	0.94	0.00
PSJ	0.03	0.01	0.00	0.00	0.00	0.00	0.01	0.00	0.95

### Historical versus recent dispersal

Inference of recent migration indicated low, asymmetric and bidirectional gene flow among Humboldt penguin colonies (Table 2). In contrast, historical gene flow was observed among all colonies (Table 3). In total 368 Humboldt penguins were sexed, where 190 were males and 178 were females (S5 Table), with no significant bias from expected 1 male:1 female proportion. However, females showed lower philopatry than males (S6 Table).

**Table 3:** Inference of theta (θ) of each population and historical migrate number of among Humboldt penguin population, estimated by maximum likelihood based on allele frequencies on MIGRATE software, where rows represent immigrants and columns represent emigrants. Population reference: CHI (Chiloé), PUP (Pupuya), ALG (Algarrobo), CAC (Cachagua), TIL (Tilgo), PAJ (Pajaros), CHO (Choros), CHA (Chañaral), GRA (Isla Grande), AZU (Pan de Azucar), and PSJ (Punta San Juan).

	ALG	CAC	TIL	PAJ	CHO	CHA	GRA	AZU	PSJ
ALG	0.012	17	20	24	50	43	23	9	13
CAC	12	0.064	24	11	21	23	14	11	34
TIL	17	29	0.089	23	44	50	79	19	92
PAJ	48	70	32	0.004	90	85	56	40	45
CHO	94	49	94	67	0.093	60	8	31	30
CHA	25	39	101	24	158	0.003	45	15	14
GRA	35	95	11	37	28	23	0.011	17	12
AZU	15	62	43	53	70	117	17	0.010	36
PSJ	21	180	64	94	80	63	55	10	0.097

### Demographic history

**Fig 5:**
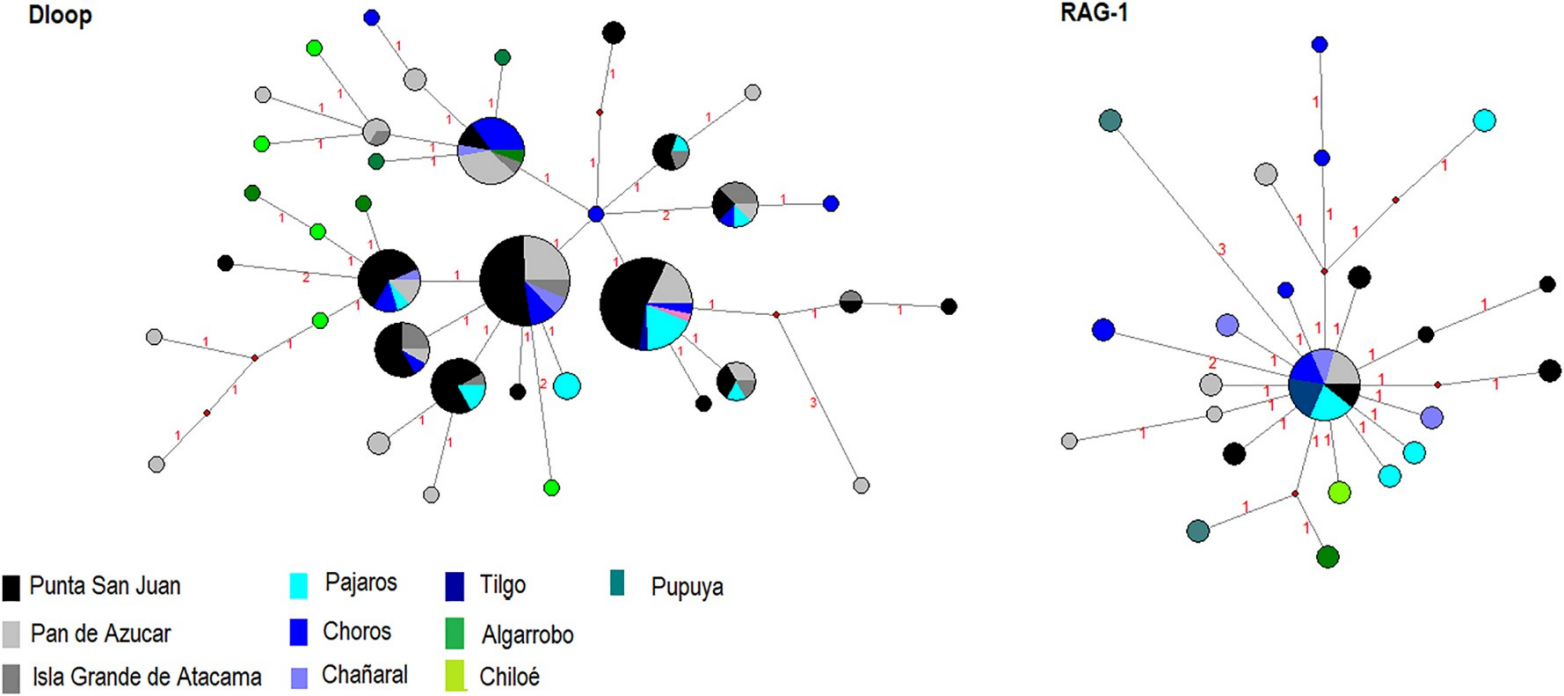
Haplotype network for the D-loop region and RAG1 from Humboldt penguin sequences. Node size corresponds to haplotype frequency.

Signatures of bottlenecks were detected at Pupuya (IAM p = 0.01), Pajaros (SMM, p < 0.01), Choros (IAM, p = 0.01; SMM, p < 0.01), Chañaral (IAM, p = 0.01; SMM, p < 0.01), Pan de Azucar (IAM, p = 0.01), and Punta San Juan (SMM, p = 0.03) (S7 Table). Furthermore, there is evidence of recent expansion of the Humboldt penguin in Punta San Juan (*Fs* = -5.87, p = 0.02) and Pan de Azucar (*Fs* = -9.31, p = 0.01; *D* = 1.63, p = 0.03) only with D-loop region (Table 1). Corroborating this expansion is the network shaped as a star topology, with few haplotypes in high frequency and several in low frequency with few mutations among them (Fig 5). Skyline plots showed coalescence around 145,000 years ago to RAG1 and 25,000 years to D-loop region (S2 Fig).

## Discussion

Our study reveals that the Humboldt penguin exhibits a clear population genetic structure along the Pacific coast of the South America, as observed in other previous studies on Humboldt penguins [22]. However, our outcome did not corroborate isolation by distance pattern [22], probably due to a gap of sampling. The present study used of several markers, a higher sample size, the distribution and the number of breeding colonies sampled throughout the whole range, and the new methods of data analysis such as the Bayesian methods applied in this study. The combination of all these appeared to overcome the effects of large population size and pulses of migration related to climate oscillations (e.g. ENSO) in the Humboldt penguin, which frequently limit the power of detection of population genetic structure. Bayesian genetic structure analyses revealed three genetic clusters in the Humboldt penguin: 1) Peru (Punta San Juan), 2) north Chile (region of Pan de Azucar and Isla Grande de Atacama), and 3) Central-South of Chile (Pajaros, Chañaral, Tilgo, Choros, Cachagua, Algarrobo) (Fig 2). This structure needs to be considered while implementing management and conservation action plans for the Humboldt penguin along the Southern Pacific coast. Also, taking into account the population data for Humboldt penguin (numbers of breeding pairs in each colony) that indicate Punta San Juan as a key colony in Peru, supporting around 3,160 breeding pairs [38]; and Pajaros, Chañaral, Tilgo and Choros together supporting around 21,700 breeding pairs (14,000 at Chañaral, 1,860 at Choros, 2,640 at Tilgo, and 1,200 at Pajaros), and Pan de Azucar with 3,000 breeding pairs. These regions need to be monitored to avoid population decline.

Although some endemic seabirds from the Humboldt Current show weak population genetic structure, such as Peruvian pelicans [5]; Peruvian boobies [12], however, other marine vertebrates, such as the Marine otter (*Lontra feline)* exhibit higher genetic differentiation from populations from Peru compared to those distributed along Chile [77]. Despite the population genetic structure among Humboldt penguin colonies, historical gene flow among several colonies was also observed (Table 3), but recent gene flow was reduced. Gene flow among seabird colonies can be associated to colder-water upwelling [12]: these areas that retain high productivity, becoming important forage sites during ENSO years. Along the Chilean coast, several upwellings have been identified, the main sites being at Antofogasta and Mejilliones, Coquimbo, and Concepcíon [16]. Thus, Humboldt penguins travel long distances to find these highly productive regions [78] to supply their diet necessity, and search main food items such as the Peruvian anchovy (*Engraulis ringens*), the Araucanian herring (*Strangomera bentincki*), the Silverside (*Odontesthes regia*), the Common hake (*Merluccius gayi*), the Inca scad (*Trachurus murphyi*), the Garfish (*Scomberesox saurus scombroides*), and the South American pilchard (*Sardinops sagax*) [79]. Therefore, the genetic structure that has been observed in the present study may result of foraging in distinct upwelling regions. It is possible that individuals from Pan de Azucar and Isla Grande de Atacama forage in Mejilliones’ colder-water upwellings and in Iquique, leading to reduced gene flow with the other colonies. This isolation of Pan de Azucar is corroborated by a known foraging radius of 35 km during the breeding season and 640 km to the north (near Iquique) during the winter [40], reducing gene flow with the other Chilean colonies. Around Isla Choros is also frequent region of colder-water upwellings favoring intense gene flow and reduced genetic structure among some islands, as Pajaros, Chañaral and Tilgo. Pajaros and Cachagua showed lower philopatry rates (0.89 and 0.86, respectively), indicating high gene flow with the other colonies. Thus, it is possible that individuals from Punta San Juan could be forage near Choros and Antofogasta in the North of the Chile, and breed there leading to gene flow among Peru and northern-central Chilean colonies, corroborating colder-water upwelling hypothesis. Vianna et al. (2014) proposed that changes in population size might have been the result of irrupt Humboldt penguin to favorable and productivity areas, moved from Punta San Juan to North of the Chile.

The population genetic structure can be explained by the species philopatric behavior that reduces gene flow. Philopatry was confirmed by assignment test based on microsatellites for all colonies of Humboldt penguins, showing individuals assignment the population origin above 86% (Table 2). Ecological data also indicate strong fidelity to natal colonies on Punta San Juan in Peru [80], and Cachagua and Algarrobo in Chile [40, 81, 82].

The evolutionary history, such as isolation, expansion or retraction of populations, affects the population genetic structure and the genetic diversity of a species. In the present study in the Humboldt penguin, the D-loop and RAG1 analyses showed a stability and recent coalescence (around 24,917 years ago to D-loop and 145,000 years ago to RAG1). Thus, it is possible that, during the glaciation when Pacific coast experienced a change in its productivity, leading to an intense reduction of the global population of the Humboldt penguin, followed by an expansion of the population after the LGM. However, the population expansion was observed only in neutrality tests to D-loop region and in network (Table 1, Fig 4). The large population size of Humboldt penguin can be masked the expansion, being important increase genomic markers to recovery demographic history. But it is not possible to discard the influence of glaciation in Humboldt penguin. Other penguin species showed the influence of climate effects on their distribution and speciation, experienced strong demographic fluctuations: during the LGM, the Gentoo penguin (*Pygoscelis papua*) maintained large effective population sizes in Antarctica and the Scotia Arc followed by an expansion [83, 84, 85], while the Adélie penguin showed two divergent lineages due to different glacial refugia in Antarctica [86, 87]. The Magellanic penguin also showed a signal of expansion after the LGM, probably due to increase of available breeding sites [3].

Penguin species have demonstrated, in general, high genetic diversity, as detected here for the Humboldt penguin, and recorded for the Magellanic penguin [3, 88], the Gentoo penguin [83, 84, 85], the Adélie penguin [84, 86, 89], and the Chinstrap penguin [9, 84]. High genetic diversity resulted from several evolutionary factors, such as large population size, low inbreeding rate, and equal sex ratio. Genetic data showed signature of drastic reduction at Pupuya, Tilgo, Pajaros, Choros, Chañaral, Pan de Azucar and Punta San Juan, indicating a bottleneck in these colonies. Despite of these bottlenecks, the Humboldt penguin has maintained high genetic diversity in all colonies.

### Considerations to conservation

Our study shows that the population structure in the Humboldt penguin can be better investigated and understood by increasing the number of markers and the sampling effort to cover the whole species’ distribution. Low sampling may not reflect real allele frequencies of the global population, showing a simplification of the genetic structure. This study of the Humboldt penguins’ population genetic structure revealed three major regions: 1) Punta San Juan in Peru, with clear genetic differences from the Chilean colonies, 2) Pan de Azucar and Isla Grande de Atacama in the North of Chile, which need a special attention as the most genetically differentiated colonies and 3) the breeding colonies from the Central-South of Chile.

Based on our results, following recommendations arise in relation to conservation initiatives. It is important to expand population genetic studies to cover other breeding colonies in Peru, to better understand the relationship between the population of Punta San Juan and the other two genetic groups detected in Chile. Chañaral and Choros are part of the Humboldt Penguin National Reserve, but the other islands (Tilgo and Pajaros)- should also be considered into this system to maintain genetic diversity and a more integral form of population management.

The Humboldt Penguin suffers from the impacts of several factors, such as the interaction with industrial fisheries (overexploited foraging species, incidental catch), the pressure from alien species (e.g. rats, *Rattus rattus* and *R*. *norvegicus*, and feral dogs) that predate on unattended eggs and chicks [90, 91], human perturbations due to tourism and guano harvesters [92], and habitat loss. Furthermore, predictions of the effects of future climate change include increases in rainfall (locally) and temperature in South America [93]. It is important to have solid monitoring systems for breeding colonies that could be affected directly by these factors, especially regarding the reduction of chick survival and reproductive success. Thus, outcomes this study help to make better decisions regarding conservation actions for this species.

## Supporting information

S1 Table Microsatellites analyzed for Humboldt penguins: Range of fragment size in base pairs (bp) (S), annealing temperature (AT), total number of alleles (N_a_), expected (H_e_) and observed heterozygosity (H_o_), Chi-Square from Hardy-Weinberg Equilibrium (HWE), probability from HWE (p). (DOCX)

S2 Table Hardy-Weinberg test to each locus from colonies of Humboldt penguin at Pacific coast. (DOCX)

S3 Table Pairwise *R*_*ST*_ based on genotypes of 10 microsatellite loci (below) and Nm based on R_ST_ (above) for Humboldt penguins. Significant values (P<0.05) are in bold. Population reference: CHI (Chiloé), PUP (Pupuya), ALG (Algarrobo), CAC (Cachagua), TIL (Tilgo), PAJ (Pajaros), CHO (Choros), CHA (Chañaral), GRA (Isla Grande), AZU (Pan de Azucar), PSJ (Punta San Juan).
(DOCX)

S4 Table Pairwise *ϕ*_*ST*_ based on mtDNA (below) and RAG1 (above) for Humboldt penguins. Significant values (P<0.05) are in bold. Population reference: CHI (Chiloé), PUP (Pupuya), ALG (Algarrobo), CAC (Cachagua), TIL (Tilgo), PAJ (Pajaros), CHO (Choros), CHA (Chañaral), GRA (Isla Grande), AZU (Pan de Azucar), PSJ (Punta San Juan).
(DOCX)

S5 Table Sex ratio of Humboldt penguin for each colony at Pacific coast. Population reference: CHI (Chiloé), PUP (Pupuya), ALG (Algarrobo), CAC (Cachagua), TIL (Tilgo), PAJ (Pajaros), CHO (Choros), CHA (Chañaral), GRA (Isla Grande), AZU (Pan de Azucar), PSJ (Punta San Juan).
(DOCX)

S6 Table Frequency of migrant male and female from first generation among colonies, and in gray proportion of philopatric rate. CAC (Cachagua), TIL (Tilgo), PAJ (Pajaros), CHO (Choros), CHA (Chañaral), GRA (Isla Grande), AZU (Pan de Azucar), PSJ (Punta San Juan).
(DOCX)

S7 Table Bottleneck summary results from SMM, IAM and TPM mutation model through Wilcoxon test, mean heterozygosity (He); mean k. Population reference: CHI (Chiloé), PUP (Pupuya), ALG (Algarrobo), CAC (Cachagua), TIL (Tilgo), PAJ (Pajaros), CHO (Choros), CHA (Chañaral), GRA (Isla Grande), AZU (Pan de Azucar), PSJ (Punta San Juan).
(DOCX)

S8 Table Results delta K´Evano implemented by Haverst web. (DOCX)

S1 Fig Number of migrants of Humboldt penguin estimated based on R_ST_ from 9 microsatellites. Population reference: CHI (Chiloé), PUP (Pupuya), ALG (Algarrobo), CAC (Cachagua), TIL (Tilgo), PAJ (Pajaros), CHO (Choros), CHA (Chañaral), GRA (Isla Grande), AZU (Pan de Azucar), PSJ (Punta San Juan).
(DOCX)

S2 Fig Skyline plot of Humboldt penguin from Pacific coast to D-loop mtDNA and RAG1 nDNA. (DOCX)

S3 Fig Discriminant function DAPC from Humboldt penguin, based on 10 microsatellites. (DOCX)

S4 Fig Bayesian STRUCTURE of the Humboldt penguin, delta K = 2 and K = 3, using admixture and no-admixture model. (DOCX)

S1 Dataset MicrossatelitesHumboldtPenguindataset- complete microsatellites data set for Humboldt penguin. (TXT)

## References

[1] Dantas GPM, Meyer D, Godinho R, Ferrand N, Morgante JS. Genetic variability in mitochondrial and nuclear genes of *Larus dominicanus* (Charadriiformes, Laridae) from the Brazilian coast. Genet Mol Biol. 2012, 35:874–885. 10.1590/S1415-47572012005000065 23271950 PMC3526097

[2] Santos FA, Morgante JS, Frere E, Millones A, Sander M, Vianna JÁ, et al Evolutionary history of the Kelp Gull (*Larus dominicanus*) in the southern hemisphere supported by multilocus evidence. J Ornithol. 2016, 157:1103–1113.

[3] Dantas GPM, Maria GC, Marasco ACM, Castro LT, Almeida VS, Santos FR, et al Demographic History and population structure of Magellanic Penguin. J. Ornithol. 2018, 159:643–655.

[4] Faria PJ, Campos FP, Branco JO, Musso CM, Morgante JS, Bruford MW. Population structure in the South American tern *Sterna hirundinacea* in the South Atlantic: two populations with distinct breeding phenologies. J Avian Biol. 2010, 41:378–387

[5] Jeyasingham WS, Taylor SA, Zavalaga CB, Simeone A, Friesen VL. Specialization to cold-water upwellings may facilitate gene flow in seabirds: new evidence from the Peruvian pelican *Pelecanus thagus* (Pelecaniformes: Pelecanidae). J. Avian Biol. 2013, 44: 297–304

[6] Younger JL, Clucas GV, Kao D, Rogers AD, Gharbi K, Hart T et al The challenges of detecting subtle population structure and its importance for the conservation of emperor penguins. Mol. Ecol. 2017, 26: 3883–3897 10.1111/mec.14172 28488293

[7] Cristofari R, Bertorelle G, Ancel A, Benazzo A, Le Maho Y, Ponganis PJ et al Full circumpolar migration ensures evolutionary unity in the Emperor penguin. Nat. Commun. 2016, 7: 11842 10.1038/ncomms11842 27296726 PMC4911614

[8] Roeder AD, Marshall RK, Mitchelson AJ, Visagathilagar T, Ritchie PA, Love DR, et al Gene flow on the ice: genetic differentiation among Adélie penguin colonies around Antarctica. Mol. Ecol. 2001, 10: 1645–1656. 11472533 10.1046/j.0962-1083.2001.01312.x

[9] Mura-Jornet I, Pimentel C, Dantas GPM, Petry MV, Gonzalez-Acuña D, Barbosa A, et al Chinstrap penguin population genetic structure: one or more populations along the Southern Ocean?. BMC Evol. Biol. 2018, 18: 90 10.1186/s12862-018-1207-0 29898661 PMC6001010

[10] Steeves TE, Anderson DJ, Friesen VL. The isthmus of Panama: a major physical barrier to gene flow in a highly mobile pantropical seabird. J. Evol. Biol. 2005a, 18: 1000–1008. 16033573 10.1111/j.1420-9101.2005.00906.x

[11] Steeves TE, Anderson DJ, Friesen VL. (2005b) A role for nonphysical barriers to gene flow in the diversification of a highly vagile seabird, the masked booby (*Sula dactylatra*). Mol. Ecol. 2005b, 14: 3877–3887 16202102 10.1111/j.1365-294X.2005.02713.x

[12] Taylor SA, Maclagan L, Anderson DJ, Friesen VL. Could specialization to cold water upwelling systems influence gene flow and population differentiation in marine organisms? A case study using the blue-footed booby, *Sula nebouxii*. J. Biogeogr. 2011, 38: 883–893.

[13] Friesen VL, Burg TM, McCoy KD. Mechanisms of population differentiation in seabirds. Mol. Ecol. 2007, 16: 1765–1785 10.1111/j.1365-294X.2006.03197.x 17444891

[14] Aravena G, Broitman B, Stenseth NC. Twelve Years of Change in Coastal Upwelling along the Central-Northern Coast of Chile: Spatially Heterogeneous Responses to Climatic Variability. PLoS ONE. 2014; 9(2): e90276 10.1371/journal.pone.0090276 24587310 PMC3938675

[15] Camus PA. Procesos regionales y fitogeografía en el Pacífico Suroriental: el efecto de ‘El Niño-Oscilación del Sur’. Rev Chil Hist. Nat. 1990, 63: 11–17.

[16] Thiel M, Macaya EC, Acuña E, Arntz WE, Bastias H et al The Humboldt current system of northern and central Chile: Oceanographic processes, ecological interactions and socioeconomic feedback. *Oceanogr*. *Mar*. *Biol*.: An Annual Review. 2007, 45: 195–344

[17] Barber RT, Chávez FP. Biological consequences of El Niño. Science 1983, 222: 1203–1210. 10.1126/science.222.4629.1203 17806711

[18] Alamo A, Bouchon M. Changes in the food and feeding of the sardine (*Sardinops sagax sagax*) during the years 1980–1984 off the Peruvian coast. J. Geophys. Res. 1987; 92: 14411–14415.

[19] Oliveira LR, Fraga LD, Majluf P. Effective population size for South American sea lions along the Peruvian coast: the survivors of the strongest El Niño event in history. J Mar Biol Assoc U.K. 2012, 92: 1835–1841.

[20] Oliveira LR, Arias-Schreiber M, Meyer D, Morgante JS. Effective population size in a bottlenecked fur seal population. Biol. Conserv. 2006, 131: 505–509.

[21] Paredes R, Zavalaga CB, Battistini G, Majluf P, McGill P. Status of the Humboldt Penguin in Peru, 1999–2000. Waterbirds 2003, 26: 129–138.

[22] Schlosser JA, Dubac JM, Garner TWJ, Arraya B, Bernal M, Simeone A, et al Evidence for gene flow differs from observed dispersal patterns in the Humboldt penguin, *Spheniscus humboldti*. Conserv Genet. 2009, 10: 839–849.

[23] Calderon L, Quintana F, Cabanne GS, Lougheed SC, Tubaro PL. Phylogeography and genetic structure of two Patagonia shag species (Aves: Phalacrocoracidae). Mol. Phylogenet. Evol. 2014, 72: 42–53 10.1016/j.ympev.2013.12.011 24418531

[24] Liebers D, Helbig AJ, De Kniff P. Genetic differentiation and phylogeography of gulls in the Larus cachinnansfuscus group (Aves, Charadriiformes). Mol Ecol. 2001, 10: 2447–2462. 11742547 10.1046/j.0962-1083.2001.01370.x

[25] Liebers D, Knijff P, Helbig AJ. The herring gull complex is not a ring species. Proc R Soc Lond B. 2004, 271:893–901 10.1098/rspb.2004.2679 PMC1691675 15255043

[26] Clapperton CM. Glacier readvances in the Andes at 12,500–10,000 YR BP: Implications for mechanism of Late–glacial climatic change. J. Quaternary Sci. 1993, 8: 197–215

[27] McCulloch RD, Bentley MJ, Purves RS, Hulton NRJ, Sugden DE, Clapperton CM. Climatic inferences from glacial and paleoecological evidence at the last glacial termination, southern South America. J. Quaternary Sci. 2000, 15: 409–417

[28] Moreno PI, Denton GH, Moreno H, Lowell TV, Putnam AE, Kaplan MR. Radiocarbon chronology of the last glacial maximum and its termination in northwestern Patagonia. Quaternary Sci. Rev. 2015, 122: 233–249.

[29] Hartl DL, Clarck AG. Principles of Population Genetics. Sinauer Associates, Sunderland; 2010 682 pp.

[30] Patterson N, Price AL, Reich D. Population structure and eigenanalysis. PLoS Genet. 2006, 2(12): e190 10.1371/journal.pgen.0020190 17194218 PMC1713260

[31] Renner SC, Suarez-Rubio M, Wiesner KR, Drogemuller C, Gockel S, Kalko EKV, et al Using multiple landscape genetic approaches to test the validity of genetic clusters in a species characterized by an isolation-by-distance pattern. 2016, Biol. J. Linn. Soc. 118:292–303

[32] Kalinowski ST. The computer program STRUCTURE does not reliably identify the main genetic clusters within species: simulations and implications for human population structure. 2011, Heredity 106: 625–632 10.1038/hdy.2010.95 20683484 PMC3183908

[33] Freer JJ, Mable BK, Clucas G, Rogers AD, Polito MJ, Dunn M, et al Limited genetic differentiation among chinstrap penguin (*Pygoscelis antarctica*) colonies in the Scotia Arc and Western Antarctic Peninsula. Polar Biol. 2015, 38:1493–1502.

[34] Williams TD. Penguins. Cambridge, British Antarctic Survey. 1991. 11pp

[35] De la Puente S, Bussaleu A, Cardeña M, Valdés-Velásquez A, Majluf P, Simeone A. Humboldt Penguin (*Spheniscus Humboldti*) In Borboroglu PG, Boersma PD (editors). Penguins; Natural History and conservation; 2013 Pp. 265–283

[36] Reyes-Arriagada R., Campos-Ellwanger P., Schatter R.P. Avifauna de Isla Guafo. Bol. Chileno Ornitol. 2009, 15: 35–43

[37] Simeone A, Hiriart-Bertrand L, Reyes-Arriagada R., Halpern M., Dubach J., Wallace R, et al Heterospecific pairing and hybridization between wild Humboldt and Magellanic penguins in southern Chile. 2009, 111: 544–550.

[38] BirdLife International. Spheniscus humboldti. (amended version published in 2016) The IUCN Red List of Threatened Species 2017: e.T22697817A111228184, 2017.

[39] Vianna JA, Cortes M, Ramos B, Sallaberry-Pincheira N, González-Acuña D, Dantas GPM, et al. Changes in abundance and distribution of Humboldt Penguin *Spheniscus Humboldti*. Mar. Ornithol. 2014, 42: 153–159

[40] Culik BM, Luna-Jorquera G. The Humboldt Penguin *Spheniscus humboldti*: a migratory bird? J. Ornithol. 1997, 138: 325–330

[41] Putz K, Raya RA, Hiriart-Bertrand L, Simeone A, Reyes-Arriagada R, Luthi B. Post-moult movements of sympatricaly breeding Humboldt and Magellanic penguins in south-central Chile. Global Ecol. Conserv. 2016, 7: 49–58

[42] Sambrook KJ, Russel DW, Sambrook J. Molecular Cloning: a Laboratory Mannual. CSHI, New York, USA 2001.

[43] Griffiths R, Double MC, Orr K., Dawon RJGA. DNA to test to sex most birds Mol. Ecol. 1998, 7: 1071–1075 9711866 10.1046/j.1365-294x.1998.00389.x

[44] Akst EP, Boersma PD, Fleischer RC. A comparison of genetic diversity between the Galápagos Penguin and the Magellanic Penguin. Conserv. Genet. 2002; 3: 375–383.

[45] Schlosser JA, Garner TWJ, Dubach JM, McElligott AG. Characterization of microsatellite loci in Humboldt penguin (*Spheniscus humboldti*) and cross-amplification in other penguin species. Mol Ecol Notes. 2003, 3: 62–64.

[46] Van-Oosterhout C, Hutchinson WF, Wills DPM, Shipley P. Micro-checker: software for identifying and correcting genotyping errors in microsatellite data. Mol. Ecol. 2004, 4: 535–538.

[47] Peakall R, Smouse PE. GenAlEx 6.5: genetic analysis in Excel. Population genetic software for teaching and research-an update. Bioinformatics. 2012, 28: 2537–2539. 10.1093/bioinformatics/bts460 22820204 PMC3463245

[48] Excoffier L, Laval G, Schneider S. Arlequin (version 3.0): An integrated software package for population genetics data analysis. J. Evolution. Bioinformatics. 2005, 1: 47–50 PMC2658868 19325852

[49] Liedloff AC. Mantel Nonparametric Test Calculator. Version 2.0. School of Natural Resource Sciences, Queensland University of Technology, Australia.1999.

[50] Pritchard JK, Stephens M, Donnelly P. Inference of population structure using multilocus genotype data. Genetics. 2000, 155: 945–959. 10835412 10.1093/genetics/155.2.945 PMC1461096

[51] Evanno G, Regnaut S, Goudet J. Detecting the number of clusters of individuals using the software structure: a simulation study. Mol. Ecol. 2005, 14: 2611*–*2620. 10.1111/j.1365-294X.2005.02553.x 15969739

[52] Jombart T. 2008 adegenet: a R package for the multivariate analysis of genetic markers. Bioinformatics 2008 24: 1403–1405. 10.1093/bioinformatics/btn129 18397895

[53] Jombart T, Devillard S., Balloux F. Discriminant analysis of principal components: a new method for the analysis of genetically structured populations. *BMC* Genetics 2010 11: 1–15. 20950446 10.1186/1471-2156-11-94 PMC2973851

[54] Piry S, Alapetite A, Cornuet JM, Paetkau D, Baudouin L, Estoup A. GENECLASS2: A Software for Genetic Assignment and First-Generation Migrant Detection. J. Hered. 2004, 95: 536–539. 10.1093/jhered/esh074 15475402

[55] Paetkau D, Calvert W, Stirling I, Strobeck C. Microsatellite analysis of population structure in Canadian polar bears. Mol. Ecol. 1995, 4: 347–354. 7663752 10.1111/j.1365-294x.1995.tb00227.x

[56] Rannala B, Mountain JL. Detecting immigration by using multilocus genotypes. PNAS. 1997, 94: 9197–9201. 10.1073/pnas.94.17.9197 9256459 PMC23111

[57] Paetkau D, Slade R, Burden M, Estoup A. Genetic assignment methods for the direct, real-time estimation of migration rate: a simulation-based exploration of accuracy and power. Mol. Ecol. 2004, 13: 55–65. 14653788 10.1046/j.1365-294x.2004.02008.x

[58] Ayres M, Ayres-Junior M, Ayres DL, Santos AS. **BioEstat 5.0**: aplicações estatísticas nas áreas das ciências biológicas e médicas. Belém: MCT; IDSM; CNPq, 2007, 364 p.

[59] Beerli P, Palczewski M. Unified framework to evaluate panmixia and migration direction among multiple sampling locations. Genetics. 2010, 185:313–326 10.1534/genetics.109.112532 20176979 PMC2870966

[60] Cornuet JM, Luikart G. Description and Power Analysis of two tests for detecting recent population bottlenecks from allele frequency data. Genetics. 1996, 144:2001–2014. 8978083 10.1093/genetics/144.4.2001 PMC1207747

[61] Piry S, Luikart G, Cornuet JM. BOTTLENECK: a computer program for detecting recent reductions in the effective population size using allele frequency data. J. Hered. 1999, 90: 502–503.

[62] Di Rienzo A, Peterson AC, Garza JC, Valdez AM, Slatkin M, Freimer NB. Mutational processes of simple-sequence repeat loci in human populations. PNAS. 1994, 91: 3166–3170. 10.1073/pnas.91.8.3166 8159720 PMC43536

[63] Roeder AD, Ritchie PA, Lambert DM. New markers for penguins. Conserv Genet. 2002, 3: 341–344.

[64] Growth JG, Barrowclough GF. Basal divergences in birds and phylogenetics utility of the nuclear RAG1 gene. Mol. Phylogenet. Evol. 1999, 12: 115–123 10.1006/mpev.1998.0603 10381315

[65] Hall TA. BioEdit: A user-friendly biological sequence alignment editor and analysis program for Windows 95/98/NT. Nucleic. Acids. Symp Ser. 2001, 41:95–98.

[66] Stephens M, Smith NJ, Donnelly P.A new statistical method for haplotype reconstruction from population data. Am J Hum Genet. 2001, 73:1162–1169 10.1086/319501 PMC1275651 11254454

[67] Librado P, Rozas J. DnaSP v.5: A Software for comprehensive analysis of DNA polymorphism data. Bioinformatics. 2009, 25: 1451–1452. 10.1093/bioinformatics/btp187 19346325

[68] Bandelt HJ, Forster P, Röhl A. Median-joining networks for inferring intraspecific phylogenies. Mol. Biol. Evol. 1999, 16: 37–48. 10.1093/oxfordjournals.molbev.a026036 10331250

[69] Tajima F. Statistical method for testing the neutral mutation hypothesis by DNA polymorphism. Genetics. 1989, 123: 585–595. 2513255 10.1093/genetics/123.3.585 PMC1203831

[70] Fu Y-X. Statistical test of Neutrality of mutations against population growth, hitchhiking and background selection. Genetics. 1997, 147: 915–925 9335623 10.1093/genetics/147.2.915 PMC1208208

[71] Ramos-Onsins SE, Rozas J. Statistical properties of new neutrality tests against population growth. Mol. Biol. Evol. 2002, 19: 2092–2100. 10.1093/oxfordjournals.molbev.a004034 12446801

[72] Posada D, Cradall KA. Modeltest: testing the model of DNA substitution. Bioinformat. Appl. Note 1998, 14: 817–818. 10.1093/bioinformatics/14.9.817 9918953

[73] Heled J, Drummond AJ. Bayesian inference of population size history from multiple loci. BMC Evol. Biol.2008, 8: 289 10.1186/1471-2148-8-289 18947398 PMC2636790

[74] Drummond AJ, Rambaut A. BEAST: Bayesian evolutionary analysis by sampling trees. BMC Evol. Biol. 2007, 7: 214 10.1186/1471-2148-7-214 17996036 PMC2247476

[75] Millar CD, Dodd A, Anderson J, Gibb GC, Ritchie PA, Baroni C, et al Mutation and evolutionary Rates in Adélie Penguins from the Antarctic. PLoS Genet. 2008, 4: e1000209 10.1371/journal.pgen.1000209 18833304 PMC2546446

[76] Zhang G, Li C, Li Q, Li B, Larkin DM, Lee C, et al (2014). Comparative genomics reveals insights into avian genome evolution and adaptation. *Science*. 2014, 346 (6215): 1311–1320. 10.1126/science.1251385 25504712 PMC4390078

[77] Axelsson E, Smith NGC, Sundstrom H, Berlin S, Ellegren H. Male-biased mutation rate and divergence in autosomal, Z-linked and W-linked introns of chicken and turkey. Mol. Biol. Evol. 2004; 21: 1538–1547. 10.1093/molbev/msh157 15140948

[78] Vianna JA, Ayerdi P, Medina-Vogel G, Mangel JC, Zeballos H, Apaza M, et al Phylogeography of the marine otter (*Lontra felina*): historical and contemporary factors determining its distribution. J. Hered. 2010, 101: 676–689. 10.1093/jhered/esq088 20688888

[79] Culik B, Hennicke J, Martin T. Humboldt Penguins outmanoeuvring El Niño. The J Exp. Biol. 2000, 203: 2311–2311. 10887069 10.1242/jeb.203.15.2311

[80] Herling C, Culik BM, Hennicke JC. Diet of the Humboldt penguin (*Spheniscus humboldti*) in northern and southern Chile. Mar. Biol. 2005, 147: 13–25

[81] Araya B, Garland D, Espinoza G, Sanhuesa A, Simeone AR, Teare A et al. Population and habitat viability assessment for the Humboldt penguin (Spheniscus humboldti), Final Report. IUCN/SSC Conservation Breeding Specialist Group, Apple Valley, MN; 2000.

[82] Wallace RS, Grzybowski K, Diebold E, Michaels MG, Teare JA, Willis MJ. Movements of Humboldt Penguins from a breeding colony in Chile. Waterbirds 1999, 22: 441–444.

[83] Peña MF., Poulin E, Dantas GPM, González-Acuña D, Petry MV, Vianna JA. Have Historical Climate Changes Affected Gentoo Penguin (Pygoscelis papua) Populations in Antarctica? PLoS ONE 2014, 9: e95375 10.1371/journal.pone.0095375 24759777 PMC3997368

[84] Clucas GV, Dunn MJ, Dyke G, Emslie SD, Naveen R, Polito MJ, et al A reversal of fortunes: climate change “winners” and “losers” in Antarctic Peninsula penguins. Sci. Rep., 2014, 4:4024. 24865774 10.1038/srep05024 PMC4034736

[85] Vianna JA, Noll D, Dantas GPM, Petry MV, Barbosa A, Gonzalez-Acuña D, et al Marked phylogeographic structure of Gentoo penguin reveals an ongoing diversification process along the Southern Ocean. Mol. Phylogenet. Evol. 2017, 107: 486–498. 10.1016/j.ympev.2016.12.003 27940333

[86] Ritchie PA, Millar CD, Gibb GC, Baroni C, Lambert DM. Ancient DNA enables timing of the Pleistocene origin and Holocene expansion of two Adélie penguin lineages in Antarctica. Mol Biol Evol. 2004, 21: 240–248. 10.1093/molbev/msh012 14595092

[87] Younger J, Emmerson L, Southwell C, Lelliott P, Miller K. Proliferation of East Antarctic Adélie penguins in response to historical deglaciation. BMC Evol. Biol. 2015, 15:236 10.1186/s12862-015-0502-2 26577544 PMC4650495

[88] Bouzat JL, Walker BG, Boersma PD. Regional Genetic Structure in the Magellanic Penguin (*Spheniscus magellanicus*) suggests Metapopulation dynamic. Auk 2009, 126: 326–334.

[89] Gorman KB., Talbot SL, Sonsthangen SA, Sage GK, Gravely MC, Fraser WR et al Population genetic structure and gene flow of Adélie penguins (*Pygoscelis adeliae*) breeding throughout the western Antarctic Peninsula. Antarctic Science. 2017, 10.1017/S0954102017000293

[90] Simeone A, Luna-Jorquera G. Estimating rat predation on Humboldt Penguin colonies in north-central Chile. J. Ornith. 2012, 153: 1079–1085

[91] Simeone A, Bernal M. Effects of Habitat Modification on Breeding Seabirds: A Case Study in Central Chile Waterbirds. 2000, 23: 449–456

[92] Ellenberg U, Mattern T, Seddon PJ, Luna_Jorquera G. Physiological and reproductive consequences of human disturbance in Humboldt Penguins: the need for species-specific visitor management. Biol. Conserv. 2006, 133: 95–106.

[93] IPCC International Pannel Climate Change: The Physical Science Basis. Contribution of Working Group I to the Fifth Assessment Report of the Intergovernmental Panel on Climate Change (eds Stocker T. et al.); 2013. pp. 1535. Cambridge University Press, New York.

