# Supplementary material for: Uncovering population structure in the Humboldt penguin (Spheniscus humboldti) along the Pacific coast at South America
Source: PLoS One. 2019 May 10;14(5):e0215293. doi: 10.1371/journal.pone.0215293 (PMC6510429; doi:10.1371/journal.pone.0215293)
Supplement: S1 Table — (DOCX) [file pone.0215293.s001.docx]

**Supplementary material**

S1 Table: Microsatellites analyzed for Humboldt Penguins: range of fragment size in base pairs (bp) (S), annealing temperature (AT), total number of alleles (N_a_), expected (H_e_) and observed heterozygosity (H_o_), Chi-Square from Hardy-Weinberg Equilibrium (HWE), probability from HWE (p)

| **Locus** | **Size (bp)** | **AT (°C)** | **N_a_** | **H_e_** | **Ho** | **HWE** | **p** |
| --- | --- | --- | --- | --- | --- | --- | --- |
| **Sh2Ca21** | 92-132 | 60 | 15 | **0.69** | **0.61** | **50.20** | **0.006** |
| **Sh1Ca12** | 100-152 | 60 | 22 | 0.84 | 0.88 | 37.70 | 0.772 |
| **Sh1Ca9** | 112-150 | 61 | 18 | 0.72 | 0.67 | 9.52 | 0.483 |
| **Sh1Ca16** | 90-142 | 58 | 21 | 0.79 | 0.75 | 10.11 | 0.431 |
| **Sh1Ca17** | 93-121 | 59 | 15 | 0.82 | 0.79 | 20.00 | 0.521 |
| **Sh2Ca12** | 87-137 | 60 | 19 | 0.74 | 0.65 | 9.35 | 0.498 |
| **Sh2Ca31** | 107-129 | 56 | 13 | 0.79 | 0.83 | 3.66 | 0.722 |
| **Sh2Ca49** | 100-126 | 56 | 13 | 0.81 | 0.80 | 5.62 | 0.466 |
| **Sh2Ca40** | 83-131 | 56 | 23 | 0.83 | 0.77 | 8.34 | 0.909 |
| **Sh2Ca55** | 90-132 | 56 | 19 | 0.70 | 0.62 | 20.29 | 0.161 |
| **Sh2Ca58** | 102-116 | 56 | 8 | 0.56 | 0.62 | 1.50 | 0.221 |
| **G2-2** | 372-398 | 54 | 14 | 0.81 | 0.83 | 29.72 | 0.760 |
| **M1-11** | 93-133 | 60 | 9 | 0.54 | 0.25 | 0.66 | 0.416 |
| **Mean** | - | - | - | 0.74 | 0.69 |  |  |

H_e_ and H_o_ in bold are the locus that showed bias to HWE (p<0.05)
