# Supplementary material for: Uncovering population structure in the Humboldt penguin (Spheniscus humboldti) along the Pacific coast at South America
Source: PLoS One. 2019 May 10;14(5):e0215293. doi: 10.1371/journal.pone.0215293 (PMC6510429; doi:10.1371/journal.pone.0215293)
Supplement: S2 Table — (DOCX) [file pone.0215293.s002.docx]

**Supplementary material**

S2 Table.: Hardy-Weinberg test to each locus from colonies of Humboldt Penguin at Pacific Coast

| **Locus** | **Chiloé** |  | **Pupuya** |  | **Algarrobo** | | **Cachagua** | |
| --- | --- | --- | --- | --- | --- | --- | --- | --- |
| **Sh2Ca21** | 5,000 | 0,172 | 1,653 | 0,949 | 17,469 | 0,065 | **74,729** | **0,000** |
| **Sh1Ca12** | 10,833 | 0,764 | 22,556 | 0,755 | **27,000** | **0,003** | 19,200 | 0,892 |
| **Sh1Ca9** | 15,000 | 0,823 | 14,280 | 0,161 | **48,000** | **0,011** | 9,175 | 1,000 |
| **Sh1Ca16** | 12,778 | 0,236 | 8,431 | 0,587 | 29,000 | 0,413 | 50,000 | 0,281 |
| **Sh1Ca17** | 15,000 | 0,451 | 24,500 | 0,655 | 35,000 | 0,170 | 36,736 | 0,435 |
| **Sh2Ca31** | 15,000 | 0,451 | 2,722 | 0,843 | 49,000 | 0,073 | 13,306 | 0,207 |
| **Sh2Ca49** | 10,000 | 0,440 | 7,933 | 0,635 | 28,000 | 0,140 | 53,896 | 0,171 |
| **Sh2Ca40** | 20,556 | 0,486 | 29,750 | 0,375 | 34,000 | 0,564 | **135,643** | **0,024** |
| **Sh2Ca55** | 10,200 | 0,116 | 10,111 | 0,431 | 11,250 | 0,338 | 28,688 | 0,122 |
| **Sh2Ca58** | 5,000 | 0,172 | 2,160 | 0,142 | **21,240** | **0,002** | **48,909** | **0,000** |
| **G2-2** | 14,167 | 0,513 | 18,000 | 0,263 | 19,688 | 0,184 | 33,519 | 0,217 |
| **Sh2Ca12** | 20,000 | 0,521 | 18,222 | 0,251 | 22,063 | 0,106 | **77,800** | **0,023** |
| **M1-11** | **10,000** | **0,019** | 0,426 | 0,514 | **27,184** | **0,002** | **35,417** | **0,000** |
| **Locus** | **Tilgo** |  | **Pajaros** |  | **Choros** |  | **Chañaral** |  |
| **Sh2Ca21** | **86,559** | **0,000** | **151,658** | **0,000** | **103,738** | **0,000** | 31,403 | 0,067 |
| **Sh1Ca12** | **232,340** | **0,000** | **191,072** | **0,000** | 115,421 | 0,899 | **167,444** | **0,000** |
| **Sh1Ca9** | **63,424** | **0,036** | **120,520** | **0,000** | **221,828** | **0,000** | **121,591** | **0,000** |
| **Sh1Ca16** | 69,393 | 0,955 | **157,888** | **0,012** | 130,979 | 0,606 | 67,891 | 0,413 |
| **Sh1Ca17** | **78,062** | **0,022** | **117,859** | **0,000** | **182,142** | **0,000** | **90,461** | **0,000** |
| **Sh2Ca31** | 37,376 | 0,406 | **122,568** | **0,000** | **89,773** | **0,002** | 68,012 | 0,112 |
| **Sh2Ca49** | **134,059** | **0,000** | **120,517** | **0,000** | **123,656** | **0,000** | 44,335 | 0,500 |
| **Sh2Ca40** | 101,980 | 0,565 | 74,831 | 0,581 | **166,242** | **0,003** | 143,482 | 0,314 |
| **Sh2Ca55** | 29,760 | 0,961 | **218,281** | **0,000** | **129,048** | **0,000** | 25,416 | 0,906 |
| **Sh2Ca58** | **8,707** | **0,033** | **111,075** | **0,000** | **172,550** | **0,000** | 1,579 | 0,664 |
| **G2-2** | 43,783 | 0,523 | 80,499 | 0,108 | **84,061** | **0,007** | **126,913** | **0,000** |
| **Sh2Ca12** | 39,455 | 0,705 | **150,583** | **0,000** | **311,286** | **0,000** | 17,918 | 0,267 |
| **M1-11** | **100,038** | **0,000** | **184,628** | **0,000** | **180,826** | **0,000** | **144,155** | **0,000** |
| **Locus** | Isl. Gde de Atacama | | Pan de Azucar | | Pta San Juan | |  |  |
| **Sh2Ca21** | 18,689 | 0,605 | 23,433 | 0,711 | **117,413** | **0,000** |  |  |
| **Sh1Ca12** | 29,201 | 0,402 | **122,132** | **0,016** | **264,801** | **0,000** |  |  |
| **Sh1Ca9** | **52,943** | **0,000** | **143,406** | **0,000** | **160,573** | **0,000** |  |  |
| **Sh1Ca16** | 18,505 | 0,617 | **64,032** | **0,003** | **158,698** | **0,001** |  |  |
| **Sh1Ca17** | 15,167 | 0,977 | 15,854 | 0,999 | **203,702** | **0,000** |  |  |
| **Sh2Ca31** | 17,833 | 0,660 | 37,420 | 0,782 | **100,574** | **0,000** |  |  |
| **Sh2Ca49** | 23,740 | 0,695 | 31,812 | 0,668 | **288,355** | **0,000** |  |  |
| **Sh2Ca40** | 25,791 | 0,585 | **130,716** | **0,000** | **200,709** | **0,006** |  |  |
| **Sh2Ca55** | 46,850 | 0,106 | **109,542** | **0,000** | **170,098** | **0,000** |  |  |
| **Sh2Ca58** | 1,170 | 0,279 | 2,414 | 0,120 | **30,541** | **0,000** |  |  |
| **G2-2** | 24,122 | 0,935 | 45,170 | 0,141 | 95,093 | 0,364 |  |  |
| **Sh2Ca12** | 9,660 | 0,471 | 33,828 | 0,572 | **189,051** | **0,000** |  |  |
| **M1-11** | 1,041 | 0,308 | 0,467 | 0,495 | **338,909** | **0,000** |  |  |
