# Supplementary material for: Uncovering population structure in the Humboldt penguin (Spheniscus humboldti) along the Pacific coast at South America
Source: PLoS One. 2019 May 10;14(5):e0215293. doi: 10.1371/journal.pone.0215293 (PMC6510429; doi:10.1371/journal.pone.0215293)
Supplement: S3 Table — Significant values (P<0.05) are in bold. Population reference: CHI (Chiloé), PUP (Pupuya), ALG (Algarrobo), CAC (Cachagua), TIL (Tilgo), PAJ (Pajaros), CHO (Choros), CHA (Chañaral), GRA (Isla Grande), AZU (Pan de Azucar), PSJ (Punta San Juan). (DOCX) [file pone.0215293.s003.docx]

**Supplementary material**

Table S3: Pairwise *R_ST_* based on genotypes of 10 microsatellite loci (below) for Humboldt Penguins. Significant values trough Bofferroni correction (P<0.05) are in bold; p value showed in superior matrix. Population reference: CHI (Chiloé), PUP (Pupuya), ALG (Algarrobo), CAC (Cachagua), TIL (Tilgo), PAJ (Pajaros), CHO (Choros), CHA (Chañaral), GRA (Isla Grande), AZU (Pan de Azucar), PSJ (Punta San Juan)

|  | PSJ | AZU | GRA | CHA | CHO | PAJ | TIL | CAC | ALG | PUP | CHI |
| --- | --- | --- | --- | --- | --- | --- | --- | --- | --- | --- | --- |
| PSJ |  | >0.001 | >0.001 | 0.027 | 0.009 | >0.001 | 0.050 | >0.001 | 0.001 | 0.650 | 0.873 |
| AZU | **0.042** |  | 0.666 | >0.001 | >0.001 | >0.001 | >0.001 | >0.001 | >0.001 | 0.009 | 0.054 |
| GRA | **0.011** | 0.002 |  | >0.001 | >0.001 | >0.001 | >0.001 | >0.001 | >0.001 | 0.009 | 0.018 |
| CHA | **0.015** | **0.013** | **0.001** |  | 0.792 | 0.070 | >0.001 | 0.099 | 0.009 | 0.054 | 0.566 |
| CHO | **0.020** | **0.025** | **0.015** | 0.004 |  | 0.720 | 0.216 | 0.009 | 0.108 | 0.027 | 0.045 |
| PAJ | **0.027** | **0.048** | **0.031** | **0.010** | 0.007 |  | 0.702 | 0.018 | 0.054 | 0.036 | 0.009 |
| TIL | **0.027** | **0.087** | **0.068** | **0.036** | 0.014 | 0.004 |  | >0.001 | >0.001 | 0.117 | 0.478 |
| CAC | **0.046** | **0.098** | **0.039** | 0.026 | **0.025** | **0.017** | **0.052** |  | 0.045 | 0.153 | 0.369 |
| ALG | **0.088** | **0.158** | **0.103** | **0.069** | 0.088 | **0.061** | **0.126** | **0.066** |  | 0.018 | 0.342 |
| PUP | 0.042 | **0.059** | **0.088** | 0.004 | **0.035** | **0.045** | 0.071 | 0.078 | **0.300** |  | 0.711 |
| CHI | 0.004 | **0.001** | **0.001** | 0.002 | **0.029** | **0.012** | 0.026 | 0.033 | 0.023 | 0.073 |  |
