# Supplementary material for: Uncovering population structure in the Humboldt penguin (Spheniscus humboldti) along the Pacific coast at South America
Source: PLoS One. 2019 May 10;14(5):e0215293. doi: 10.1371/journal.pone.0215293 (PMC6510429; doi:10.1371/journal.pone.0215293)
Supplement: S4 Table — Significant values (P<0.05) are in bold. Population reference: CHI (Chiloé), PUP (Pupuya), ALG (Algarrobo), CAC (Cachagua), TIL (Tilgo), PAJ (Pajaros), CHO (Choros), CHA (Chañaral), GRA (Isla Grande), AZU (Pan de Azucar), PSJ (Punta San Juan). (DOCX) [file pone.0215293.s004.docx]

**Supplementary material**

S4 Table.: Pairwise *ϕ_ST_* based on mtDNA (below) and RAG1 (above) for Humboldt Penguins. Significant values (P<0.05) are in bold. Population reference: CHI (Chiloé), PUP (Pupuya), ALG (Algarrobo), CAC (Cachagua), TIL (Tilgo), PAJ (Pajaros), CHO (Choros), CHA (Chañaral), GRA (Isla Grande), AZU (Pan de Azucar), PSJ (Punta San Juan)

|  | PSJ | AZU | GRA | CHA | CHO | PAJ | TIL | CAC | ALG | PUP | CHI |
| --- | --- | --- | --- | --- | --- | --- | --- | --- | --- | --- | --- |
| PSJ |  | **0.010** | - | **0.113** | **0.124** | 0.070 | >0.001 | **0.312** | **0.484** | - | 0.275 |
| AZU | 0.005 |  | - | **0.124** | **0.063** | 0.065 | >0.001 | **0.341** | **0.542** | - | **0.333** |
| GRA | 0.009 | 0.016 |  | - | - | - | - | - | - | - | - |
| CHA | 0.019 | >0.001 | 0.047 |  | **0.145** | 0.126 | 0.111 | **0.358** | 0.684 | - | 0.510 |
| CHO | **0.047** | 0.005 | 0.040 | >0.001 |  | 0.091 | 0.020 | **0.304** | 0.479 | - | 0.279 |
| PAJ | >0.001 | >0.001 | 0.009 | 0.017 | 0.038 |  | >0.001 | **0.330** | 0.518 | - | 0.310 |
| TIL | >0.001 | >0.001 | >0.001 | >0.001 | 0.051 | >0.001 |  | 0.333 | 1.000 | - | 1.000 |
| CAC | **-** | **-** | **-** | **-** | **-** | **-** | **-** |  | **0.344** | - | 0.344 |
| ALG | 0.085 | 0.002 | 0.047 | >0.001 | >0.001 | 0.046 | >0.001 | - |  | - | 1.000 |
| PUP | >0.001 | >0.001 | 0.008 | >0.001 | >0.001 | >0.001 | >0.001 | - | >0.001 |  | - |
| CHI | **-** | **-** | **-** | - | - | - | - | - | - | - |  |
