# Supplementary material for: Uncovering population structure in the Humboldt penguin (Spheniscus humboldti) along the Pacific coast at South America
Source: PLoS One. 2019 May 10;14(5):e0215293. doi: 10.1371/journal.pone.0215293 (PMC6510429; doi:10.1371/journal.pone.0215293)
Supplement: S5 Table — Population reference: CHI (Chiloé), PUP (Pupuya), ALG (Algarrobo), CAC (Cachagua), TIL (Tilgo), PAJ (Pajaros), CHO (Choros), CHA (Chañaral), GRA (Isla Grande), AZU (Pan de Azucar), PSJ (Punta San Juan). (DOCX) [file pone.0215293.s005.docx]

**Supplementary material**

S5 Table.:Sex ratio of Humboldt Penguin for each colony at Pacific Coast. Population reference: CHI (Chiloé), PUP (Pupuya), ALG (Algarrobo), CAC (Cachagua), TIL (Tilgo), PAJ (Pajaros), CHO (Choros), CHA (Chañaral), GRA (Isla Grande), AZU (Pan de Azucar), PSJ (Punta San Juan)

| **Colony** | **N** | **Male** | **Female** | **Male/Female** | ᵪ² | **p** |
| --- | --- | --- | --- | --- | --- | --- |
| CHI | 4 | 1 | 3 | 0.33 | 1.04 | 0.30 |
| PUP | 15 | 9 | 6 | 1.50 | 0.66 | 0.43 |
| ALG | 6 | 2 | 4 | 0.50 | 0.66 | 0.40 |
| CAC | 13 | 8 | 5 | 1.60 | 0.69 | 0.40 |
| TIL | 38 | 22 | 16 | 1.37 | 0.94 | 0.33 |
| PAJ | 62 | 31 | 31 | 1.00 | 0.00 | 1.00 |
| CHO | 56 | 25 | 31 | 0.80 | 0.64 | 0.42 |
| CHA | 43 | 30 | 13 | 2.30 | 6.72 | 0.01 |
| GRA | 12 | 6 | 6 | 1.00 | 0.00 | 1.00 |
| AZU | 36 | 20 | 16 | 1.25 | 0.44 | 0.50 |
| PSJ | 83 | 36 | 47 | 0.76 | 1.44 | 0.22 |
| total | 368 | 190 | 178 | 1.06 |  |  |
