# Supplementary material for: Uncovering population structure in the Humboldt penguin (Spheniscus humboldti) along the Pacific coast at South America
Source: PLoS One. 2019 May 10;14(5):e0215293. doi: 10.1371/journal.pone.0215293 (PMC6510429; doi:10.1371/journal.pone.0215293)
Supplement: S6 Table — CAC (Cachagua), TIL (Tilgo), PAJ (Pajaros), CHO (Choros), CHA (Chañaral), GRA (Isla Grande), AZU (Pan de Azucar), PSJ (Punta San Juan). (DOCX) [file pone.0215293.s006.docx]

**Supplementary material**

S6: Frequency of migrant male and female from first generation among colonies, and in gray proportion of philopatric rate. CAC (Cachagua), TIL (Tilgo), PAJ (Pajaros), CHO (Choros), CHA (Chañaral), GRA (Isla Grande), AZU (Pan de Azucar), PSJ (Punta San Juan)

| Male | | | | | | | | | |
| --- | --- | --- | --- | --- | --- | --- | --- | --- | --- |
|  | CAC | TIL | PAJ | CHO | CHA | GRA | AZU | PSJ |  |
| CAC | 0.75 | - | - | - | - | 0.25 | - | - |  |
| TIL | - | 0.90 | 0.04 | - | - | 0,04 | - | - |  |
| PAJ | 0.03 | - | 0.93 | - | 0.03 | - | - | - |  |
| CHO | - | - | - | 0.92 | - | 0.08 | - | - |  |
| CHA | - | 0.06 | 0.03 | - | 0.81 | - | - | - |  |
| GRA | - | - | - | - | - | 1.00 | - | - |  |
| AZU | - | - | - | - | - | 0.05 | 0.95 | - |  |
| PSJ | - | 0.02 | 0.02 | - | - | 0.05 | - | 0.88 |  |
| Female | | | | | | | | | |

|  | CAC | TIL | PAJ | CHO | CHA | GRA | AZU | PSJ |
| --- | --- | --- | --- | --- | --- | --- | --- | --- |
| CAC | 0.60 | 0.20 | - | - | 0.20 | - | - | - |
| TIL | - | 0.87 | - | - | - | 0.06 | 0.06 | - |
| PAJ | 0.03 | - | 0.93 | - | - | 0.03 | - | - |
| CHO | - | - | - | 1.00 | - | - | - | - |
| CHA | 0.23 | - | - | - | 0.76 | - | - | - |
| GRA | - | - | - | - | - | 1.00 | - | - |
| AZU | 0.06 | - | - | - | - | 0.12 | 0.81 |  |
| PSJ | 0.04 | - | - | - | - | - | - | 0.95 |
