# Supplementary material for: Uncovering population structure in the Humboldt penguin (Spheniscus humboldti) along the Pacific coast at South America
Source: PLoS One. 2019 May 10;14(5):e0215293. doi: 10.1371/journal.pone.0215293 (PMC6510429; doi:10.1371/journal.pone.0215293)
Supplement: S7 Table — Population reference: CHI (Chiloé), PUP (Pupuya), ALG (Algarrobo), CAC (Cachagua), TIL (Tilgo), PAJ (Pajaros), CHO (Choros), CHA (Chañaral), GRA (Isla Grande), AZU (Pan de Azucar), PSJ (Punta San Juan). (DOCX) [file pone.0215293.s007.docx]

**Supplementary material**

S7 Table: Bottleneck summary results from SMM, IAM and TPM mutation model through Wilcoxon test, mean heterozygosity (He); mean k. Population reference: CHI (Chiloé), PUP (Pupuya), ALG (Algarrobo), CAC (Cachagua), TIL (Tilgo), PAJ (Pajaros), CHO (Choros), CHA (Chañaral), GRA (Isla Grande), AZU (Pan de Azucar), PSJ (Punta San Juan)

| Population | mean_k | mean_He | p_IAM | p_TPM | p_SMM |
| --- | --- | --- | --- | --- | --- |
| CHI | 5.44 | 0.834 | 0.359 | 0.820 | 0.328 |
| PUP | 5.67 | 0.833 | **0.001** | **0.009** | 0.128 |
| ALG | 7.00 | 0.812 | 0.251 | 0.820 | 0.570 |
| CAC | 9.22 | 0.812 | 0.652 | 0.496 | 0.203 |
| TIL | 10.89 | 0.817 | **0.003** | 0.570 | **0.037** |
| PAJ | 11.56 | 0.803 | **0.027** | 0.820 | **0.005** |
| CHO | 12.22 | 0.824 | **0.001** | 0.734 | **0.001** |
| CHA | 10.33 | 0.798 | **0.001** | 0.570 | **0.001** |
| GRA | 7.11 | 0.756 | 0.820 | 0.496 | 0.164 |
| AZU | 8.78 | 0.784 | **0.001** | 0.250 | **0.037** |
| PSJ | 13.11 | 0.821 | **0.003** | 0.910 | **0.013** |
