# Supplementary material for: Uncovering population structure in the Humboldt penguin (Spheniscus humboldti) along the Pacific coast at South America
Source: PLoS One. 2019 May 10;14(5):e0215293. doi: 10.1371/journal.pone.0215293 (PMC6510429; doi:10.1371/journal.pone.0215293)
Supplement: S8 Table — (DOCX) [file pone.0215293.s008.docx]

**Supplementary material**

S8 Table of Results Delta K´Evano implemented by Haverst web.

| No admixture model | | | | | | |
| --- | --- | --- | --- | --- | --- | --- |
| # K | Reps | Mean LnP(K) | Stdev LnP(K) | Ln'(K) | \|Ln''(K)\| | Delta K |
| 1 | 10 | -232,130.900 | 0.2025 | NA | NA | NA |
| 2 | 10 | -223.483.600 | 317.551 | 243.240.000 | 18.070.000 | 0.569042 |
| 3 | 10 | -225.916.000 | 14.937 | 621.490.000 | 378.250.000 | 0.569042 |
| 4 | 10 | -221.231.900 | 102.954 | 225.170.000 | 223.590.000 | 253.231.855 |
| 5 | 10 | -221.216.100 | 907.978 | 1.580.000 | 177.060.000 | 1.950.047 |
| 6 | 10 | -219.429.700 | 167.847 | 178.640.000 | 67.240.000 | 4.006.041 |
| 7 | 10 | -218.315.700 | 147.990 | 111.400.000 | 120.470.000 | 8.140.441 |
| 8 | 10 | -218.406.400 | 406.959 | -9.070.000 | 0.030000 | 0.000737 |
| 9 | 10 | -218.496.800 | 1.619.630 | -9.040.000 | 121.150.000 | 0.748010 |
| 10 | 10 | -217.375.700 | 1.044.558 | 112.110.000 | NA | NA |
| Admixture model | | | | | | |
| # K | Reps | Mean LnP(K) | Stdev LnP(K) | Ln'(K) | \|Ln''(K)\| | Delta K |
| 1 | 10 | -232.130.900 | 0.4202 | NA | NA | NA |
| 2 | 10 | -223.029.400 | 76.053 | 314.700.000 | 191.020.000 | 25.116.726 |
| 3 | 10 | -226.176.400 | 21.130 | 595.450.000 | 280.750.000 | 132.866.176 |
| 4 | 10 | -221.792.600 | 67.589 | 123.680.000 | 119.660.000 | 17.704.076 |
| 5 | 10 | -221.752.400 | 495.243 | 4.020.000 | 21.300.000 | 0.430092 |
| 6 | 10 | -221.925.200 | 1.994.283 | -17.280.000 | 132.500.000 | 0.664399 |
| 7 | 10 | -220.773.000 | 940.210 | 115.220.000 | 113.020.000 | 1.202.071 |
| 8 | 10 | -220.751.000 | 2.142.707 | 2.200.000 | 116.360.000 | 0.543051 |
| 9 | 10 | -221.892.600 | 3.215.721 | -114.160.000 | 214.420.000 | 0.666787 |
| 10 | 10 | -220.890.000 | 1.126.556 | 100.260.000 | NA | NA |
