# Supplementary material for: Uncovering population structure in the Humboldt penguin (Spheniscus humboldti) along the Pacific coast at South America
Source: PLoS One. 2019 May 10;14(5):e0215293. doi: 10.1371/journal.pone.0215293 (PMC6510429; doi:10.1371/journal.pone.0215293)
Supplement: S1 Fig — Population reference: CHI (Chiloé), PUP (Pupuya), ALG (Algarrobo), CAC (Cachagua), TIL (Tilgo), PAJ (Pajaros), CHO (Choros), CHA (Chañaral), GRA (Isla Grande), AZU (Pan de Azucar), PSJ (Punta San Juan). (DOCX) [file pone.0215293.s009.docx]

**Supplementary material** **
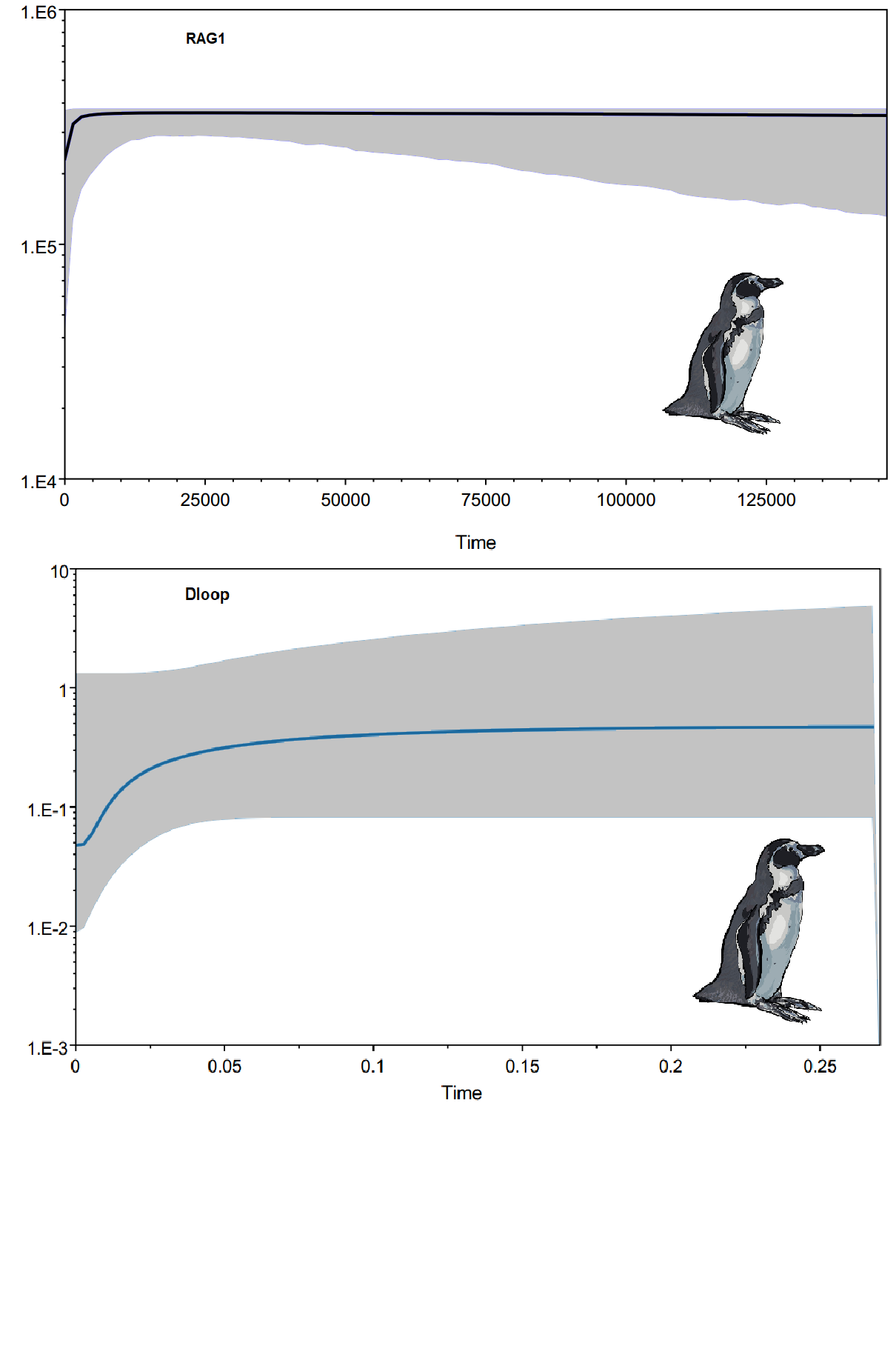
**S1 Figure:. Skyline Plot of Humboldt Penguin from Pacific coast to D-loop mtDNA and RAG1 nDNA.
