# Supplementary material for: Uncovering population structure in the Humboldt penguin (Spheniscus humboldti) along the Pacific coast at South America
Source: PLoS One. 2019 May 10;14(5):e0215293. doi: 10.1371/journal.pone.0215293 (PMC6510429; doi:10.1371/journal.pone.0215293)
Supplement: S2 Fig — (DOCX) [file pone.0215293.s010.docx]

**Supplementary material**

S2. Relationship between pairwise genetic differentiation (R_ST_) and geographic distance among Humboldt penguin colonies
